# Three-dimensional structure of a mycobacterial oligoribonuclease reveals a unique C-terminal tail that stabilizes the homodimer

**DOI:** 10.1016/j.jbc.2022.102595

**Published:** 2022-10-14

**Authors:** Pooja Badhwar, Sabab Hasan Khan, Bhupesh Taneja

**Affiliations:** 1 CSIR-Institute of Genomics and Integrative Biology (CSIR-IGIB), New Delhi, India; 2 Academy of Scientific and Innovative Research (AcSIR), Ghaziabad, India

**Keywords:** cyclic di-GMP, mycobacteria, crystal structure, structure–function, structural biology, RNase-H fold, DEDDh motif, oligoribonuclease, ANS, 8-anilino-1-naphthalene sulfonic acid, bNPP, bis-(*p*-nitrophenol) phosphate, c-di-GMP, cyclic-di-GMP, DGC, diguanylate cyclase, Eco_orn, Orn of *Escherichia coli*, EAL, glutamate-alanine-leucine, ESRF, European Synchrotron Radiation Facility, Gdm-HCl, guanidium hydrochloride, HD-GYP, histidine-aspartate-glycine-tyrosine-proline, MALLS, multiangle laser light scattering, MD, molecular dynamics, Ms_orn, Orn of *Mycobacterium smegmatis*, Mtb_orn, Orn of *Mycobacterium tuberculosis*, Ni–NTA, nickel–nitrilotriacetate, Orn, oligoribonuclease, P-cap, phosphate-cap, PDB, Protein Data Bank, PDE, phosphodiesterase, pNPP, *p*-nitrophenol phosphate, qRT–PCR, quantitative RT–PCR, SEC, size-exclusion chromatography, Vc_orn, Orn of *Vibrio cholerae*

## Abstract

Oligoribonucleases (Orns) are highly conserved DnaQ-fold 3′-5′ exoribonucleases that have been found to carry out the last step of cyclic-di-GMP (c-di-GMP) degradation, that is, pGpG to GMP in several bacteria. Removal of pGpG is critical for c-di-GMP homeostasis, as excess uncleaved pGpG can have feedback inhibition on phosphodiesterases, thereby perturbing cellular signaling pathways regulated by c-di-GMP. Perturbation of c-di-GMP levels not only affects survival under hypoxic, reductive stress, or nutrient-limiting conditions but also affects pathogenicity in infection models as well as antibiotic response in mycobacteria. Here, we have determined the crystal structure of MSMEG_4724, the Orn of *Mycobacterium smegmatis* (Ms_orn) to 1.87 Å resolution to investigate the function of its extended C-terminal tail that is unique among bacterial Orns. Ms_orn is a homodimer with the canonical RNase-H fold of exoribonucleases and conserved catalytic residues in the active site. Further examination of the substrate-binding site with a modeled pGpG emphasized the role of a phosphate cap and “3′OH cap” in constricting a 2-mer substrate in the active site. The unique C-terminal tail of Ms_orn aids dimerization by forming a handshake-like flap over the second protomer of the dimer. Our thermal and denaturant-induced unfolding experiments suggest that it helps in higher stability of Ms_orn as compared with *Escherichia coli* Orn or a C-terminal deletion mutant. We also show that the C-terminal tail is required for modulating response to stress agents *in vivo.* These results will help in further evaluating the role of signaling and regulation by c-di-GMP in mycobacteria.

Cyclic-di-GMP (bis-3′-5′ cyclic dimeric GMP or c-di-GMP) is one of the most widely distributed cyclic dinucleotide signaling molecules in bacteria (1, 2, 3, 4). It was first reported as an allosteric activator of membrane-bound cellulose synthase in *Komagataeibacter xylinus* (formerly known as *Gluconacetobacter xylinus*) in 1987 (5). Since then, c-di-GMP has been discovered as a key bacterial secondary messenger, regulating several signaling networks *viz*., motility and biofilm formation in *Pseudomonas aeruginosa* (6, 7, 8), *Pseudomonas fluorescens* (9), *Salmonella typhimurium* (10), and *Listeria monocytogenes* (11); control of cell cycle progression and development in *Caulobacter crescentus* (12, 13) and *Streptomyces veneuelae* (14) and affects virulence and pathogenicity of several animal and plant pathogens like *Vibrio cholerae* (15, 16), *Xanthomonas campesteris* (17, 18), *Klebsiella pneumoniae* (19), and *Mycobacterium tuberculosis* (20).

Alteration of c-di-GMP levels in *Mycobacterium smegmatis* affects long-term survival of the bacterium under carbon-limiting conditions (21, 22) and response to antibiotics because of modulation of lipid biosynthesis (23, 24). In *M. tuberculosis*, increased levels of c-di-GMP reduces bacterial dormancy and decreases infectivity in human THP-1 cells and mouse infection models (20). In addition, binding of c-di-GMP to EthR (a TetR-like transcription regulator) is found to enhance binding of EthR to *ethA* promoter and represses the transcription of monooxygenase EthA. This results in resistance of *M. tuberculosis* to ethionamide, a second-line antituberculosis drug, by preventing activation of the prodrug by EthA (25), further emphasizing the role of this important signaling molecule in mycobacterial cells.

In order to maintain optimal intracellular levels of c-di-GMP in the cell, a distinct set of enzymes are required. A diguanylate cyclase (DGC) that harbors a glycine-glycine-aspartate-glutamate-phenylalanine (GGDEF) domain has been shown to be required for biosynthesis of c-di-GMP in several bacteria. Degradation of c-di-GMP, may however, vary in different bacteria (1, 2, 3, 4). In *P*. *aeruginosa* (26) and *X. campesteris* (27, 28), histidine-aspartate-glycine-tyrosine-proline (HD-GYP) domain containing proteins degrade c-di-GMP fully to GMP. By contrast, in *Escherichia coli* (29), *P*. *aeruginosa* (6, 30), and *C. crescentus* (31), glutamate-alanine-leucine (EAL) domain containing phosphodiesterases (PDEs) catalyze the asymmetric hydrolysis of c-di-GMP to yield linear di-GMP (pGpG), which must be further catabolized to GMP either by HD-GYP domain containing proteins or by specific exoribonucleases termed oligoribonucleases (Orns) (32, 33, 34). In mycobacteria, a bifunctional DGC having both GGDEF and EAL domains brings about biosynthesis or degradation of c-di-GMP to pGpG through the respective domains (21, 35). Degradation of c-di-GMP to pGpG may also be affected by an associated PDE (Rv1357c) in *M. tuberculosis* (20). However, there is no HD-GYP domain containing PDE in mycobacteria, emphasizing the role of Orn to bring about the final step of degradation of pGpG to GMP.

Orns are highly conserved DnaQ-fold 3′-5' exoribonucleases with a DEDDh active site motif that degrade short 2- to 5-mer oligoribonucleotides in 3′-5' direction and release monoribonucleotides as reaction products (32, 36, 37, 38, 39). Deletion of *orn* led to accumulation of oligonucleotides in *E. coli* (40), mispriming of transcripts in *P. aeruginosa* (41) and affects the viability of bacterial cells (40). Deletion of *orn* in *P. aeruginosa* also exhibited reduced susceptibility toward antibacterial drugs (42, 43, 44) with reduced pathogenesis in mouse infection models (44). In mycobacteria, although effect of *orn* knockout has not been studied so far, *dgc* or *pde* mutant strains exhibited altered colony morphology and altered growth profile in *M. smegmatis* (21, 35) and reduced pathogenicity in *M. tuberculosis* (20).

In *M. smegmatis*, MSMEG_4724 is classified as the ortholog of Orn of *M. tuberculosis* (Rv2511), sharing an overall sequence similarity of nearly 80%. Interestingly, while bacterial Orns are usually 170 to 180 residues long, mycobacterial Orns are longer with an extended 30- to 35-residue long C-terminal tail of unknown function. However, the role of this unique extended C-terminal tail of Orn of *M. smegmatis* (Ms_orn), in maintaining the structure, function, or stability, has not been determined thus far. In this study, we have structurally characterized MSMEG_4724 to delineate the main structural characteristics of this mycobacterial Orn and its unique C-terminal tail in particular. Overall, this work shows that the C-terminal tail is required for higher stability of Ms_orn and in modulating response to stress agents *in vivo* and will help in better understanding cyclic dinucleotide–mediated signaling in mycobacteria.

## Results

### Protein purification and key sequence features

**Figure 1:**
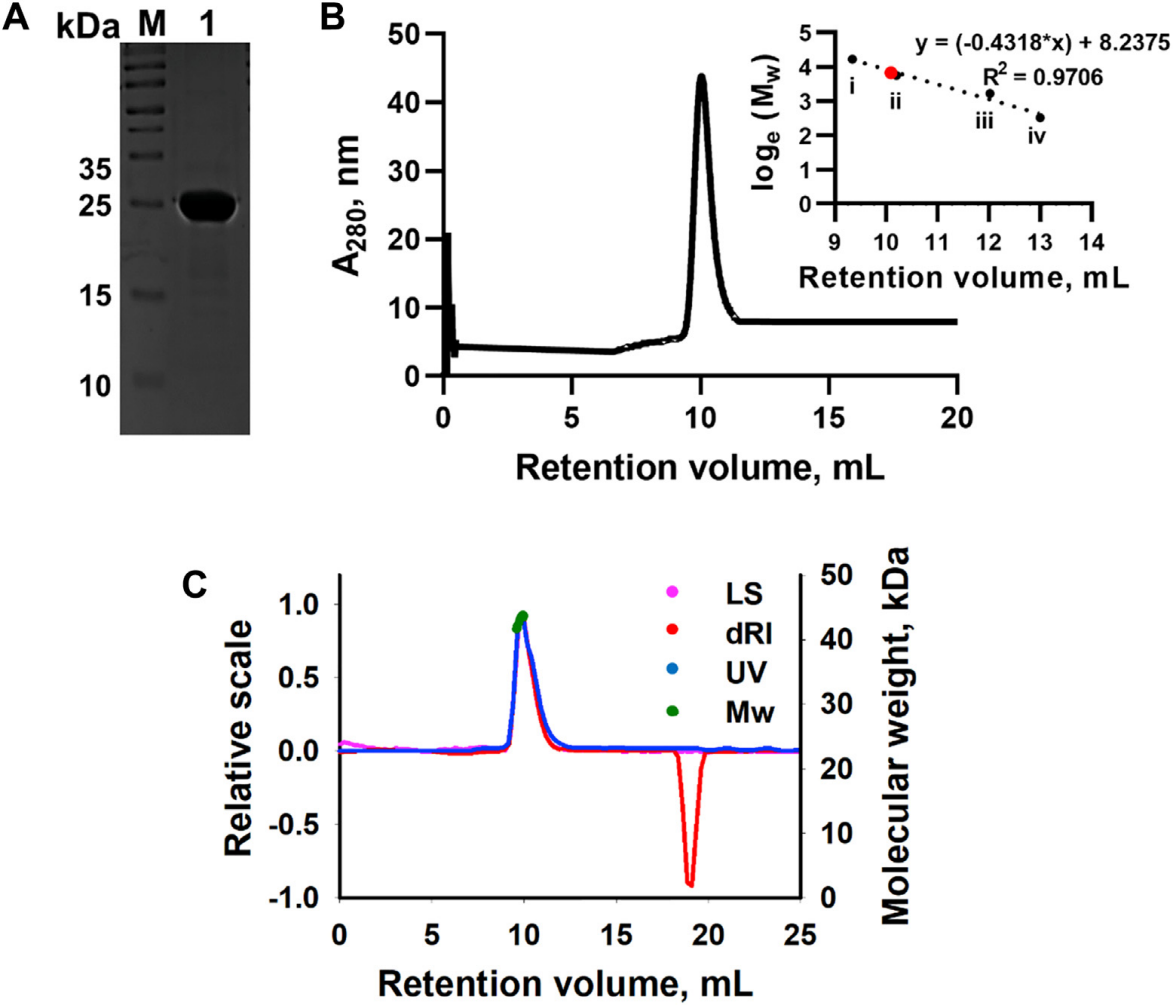
**Purification and size estimation of Ms_orn.***A*, electrophoretic profile of purified Ms_orn on a 12% SDS-PAGE indicates a band of high purity with apparent *M*_w_ of 23 kDa. *B*, SEC profile of Ms_orn on Superdex-75 column. Ms_orn eluted as a single peak at 10.1 ml, corresponding to a dimer when compared with calibrated gel filtration data of molecular weight standards (shown in *inset*) ((i) bovine serum albumin, 66 kDa; (ii) ovalbumin, 45 kDa; (iii) chymotrypsinogen, 25 kDa; (iv) cytochrome *c*, 12.5 kDa. Ms_orn is indicated in *red*). *C*, SEC–MALLS of Ms_orn showing signal peaks of LS (light scattering), dRI (differential refractive index), and UV (at 280 nm), and a calculated *M*_w_ (*green*) of 42.97 kDa. MALLS, multiangle laser light scattering; Ms_orn, Orn of *Mycobacterium smegmatis*; SEC, size-exclusion chromatography.

Ms_orn was purified to homogeneity and its purity, oligomeric state and monodispersity were confirmed on 12% SDS-PAGE, by size-exclusion chromatography (SEC) and light scattering measurements (Fig. 1). Ms_orn eluted as a single peak on a SEC column, corresponding to a dimer in solution. Light scattering measurements with SEC–multiangle laser light scattering (MALLS) confirm a homogenous monodisperse population for Ms_orn with a molar mass (*M*_w_) of 4.297 × 10^4^ g/mol (Fig. 1*C*), confirming the dimeric form of the protein in solution.

**Figure 2:**
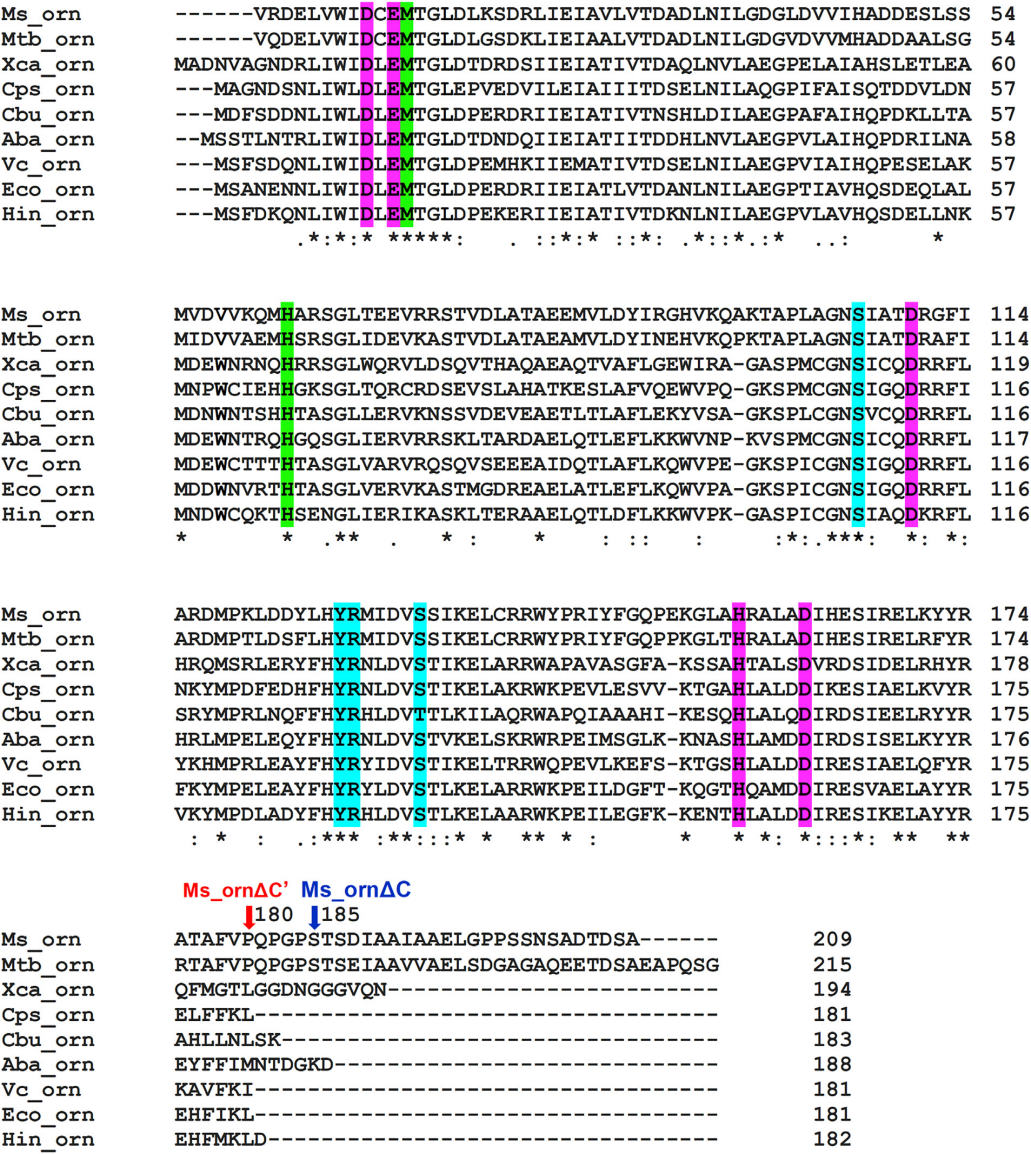
**Sequence analysis of Ms_orn**. Sequence alignment of Ms_orn and *Mycobacterium tuberculosis* orn (Mtb_orn: Rv2511) is shown with other bacterial oligoribonucleases (Orns) whose structures are available in Protein Data Bank (PDB). Sequences of Orn of *Xanthomonas campestris*, (Xca_orn; PDB ID: 2GBZ), *Colwellia psychrerythraea* (Cps_orn; PDB ID: 6A4A), *Coxiella burnetii* (Cbu_orn; PDB ID: 3TR8), *Acinetobacter baumannii* (Aba_orn; PDB ID: 5CY4), *Vibrio cholerae* (Vc_orn; PDB ID: 6N6A), *Escherichia coli* (Eco_orn; PDB ID: 2IGI), and *Haemophilus influenzae* (Hin_orn; PDB ID: 1J9A) are shown. Ms_orn shares 41.5%, 43.8%, 42.8%, 44.0%, 43.3%, 46.6%, and 44.7% sequence similarity with each bacterial Orn, respectively. Catalytic residues conserved in Orns are highlighted in *magenta*, whereas conserved 5′-phosphate cap residues (*cyan*) and “3′-OH cap” residues (*green*) are also indicated. Positions 180 and 185, as C termini of Ms_ornΔC′ and Ms_ornΔC are indicated by *red* and *blue arrows*, respectively. Ms_orn, Orn of *Mycobacterium smegmatis*.

Sequence analysis of Ms_orn using Conserved Domain Database indicated that the protein belongs to DEDDh-type DnaQ-like 3′-5′ Orn family that cleaves RNA in a metal-dependent manner. Sequence comparison of Ms_orn with the other bacterial Orns, whose structures are available in Protein Data Bank (PDB), reveals more than 40% sequence identity across the entire length of the protein sequences with high degree of conservation surrounding the catalytic DEDDh residues (Fig. 2). Significant level of conservation is also seen for residues required in substrate binding or present at dimer interface (discussed later). The C-terminal region of Orns, however, shows a lot of variability and differences in lengths across all sequences. The C-terminal region of mycobacterial Orns is much longer, with a 29-residue long C-terminal tail (residues 181–209) in Ms_orn, in particular (Fig. 2). This extended C-terminal tail of Ms_orn hence appears to be unique among bacterial Orns and any function associated with it remains to be elucidated.

### Overall structure of Ms_orn

**Figure 3:**
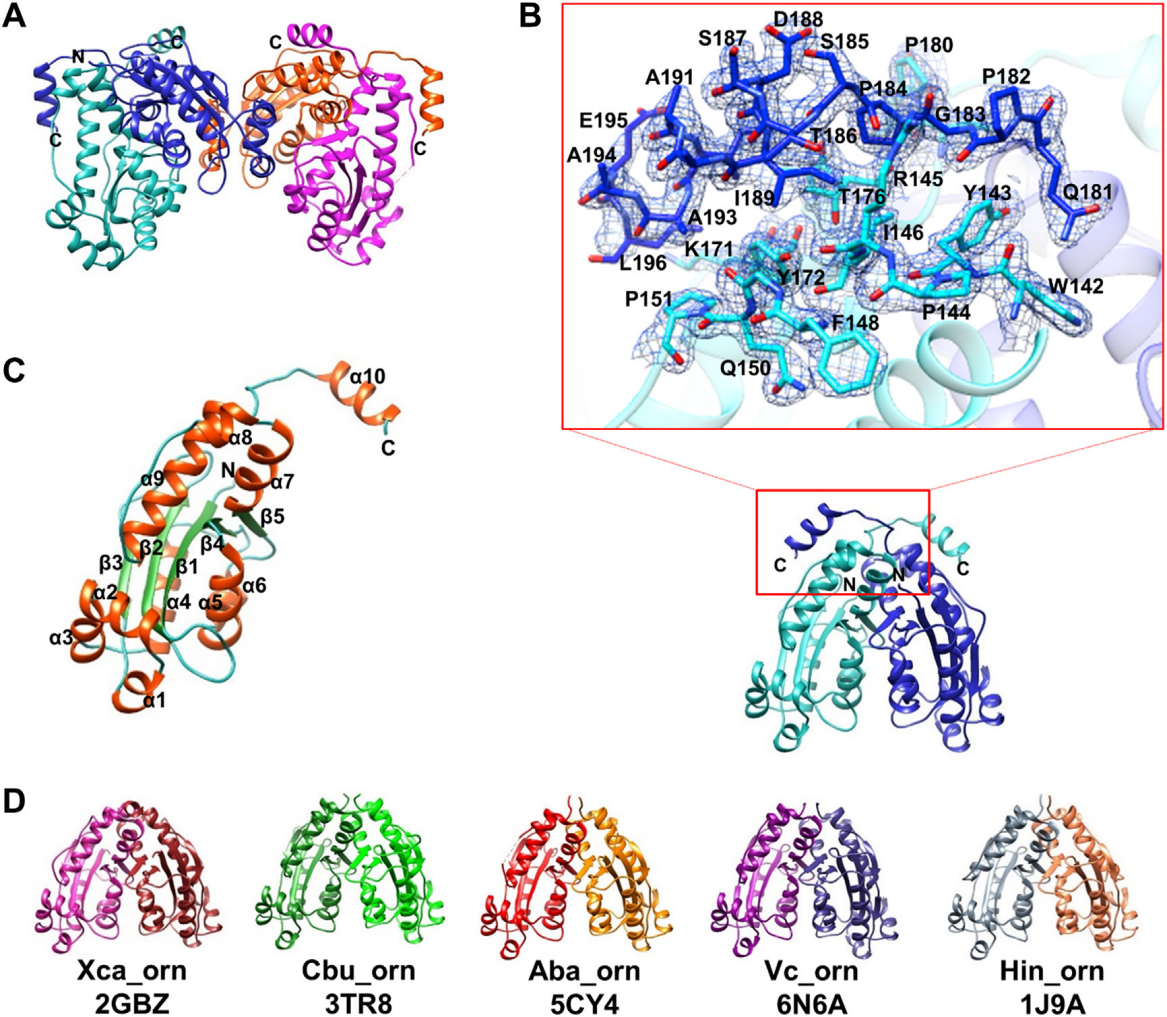
**Crystal structure of Ms_orn.***A*, two homodimers of Ms_orn (one dimer in different hues of *blue* and other in *magenta*) in side-to-side orientation in the asymmetric unit (a.s.u.). *B*, biological unit of Ms_orn homodimer with the extended C-terminal helix of one protomer packing against the other in a “handshake”-like manner. A close-up view of the C-terminal tail showing the interactions of the residues in the tail with the main domain of the other subunit is shown. A 2*F*_o_–*F*_c_ map contoured at 1.5 sigma level for the C-terminal chain is also plotted. *C*, ribbon diagram of Ms_orn protomer (chain A) showing the nine α-helices surrounding the five central β-sheets along with the extended helix, α10. *D*, homodimers of bacterial Orns (strain names and Protein Data Bank IDs are mentioned in Fig. 2) clearly indicate that the C-terminal helical flap is unique only to Ms_orn. Ms_orn, Orn of *Mycobacterium smegmatis*.

The structure of Ms_orn in apo form was determined to 1.87 Å resolution to a final *R*_work_ of 17.9% and *R*_free_ of 22.2% (Table 1). There are four molecules of Ms_orn in one asymmetric unit with two homodimers packed in a side-to-side orientation (Fig. 3*A*). PISA server (45) indicated homodimer as the probable quaternary structure for Ms_orn, which was confirmed by SEC–MALLS (Fig. 1*C*).

**Table 1:** X-ray data collection and structure refinement statistics^a^

Parameter	Ms_orn	Eco_orn
PDB ID	7WIK	7VH4
Beam source	Synchrotron	Synchrotron
	Beamline ID29	Beamline ID29
Wavelength (Å)	0.9686	0.9686
Resolution (Å)	81.07–1.87 (1.90–1.87)	59.63–2.30 (2.38–2.30)
Space group	P2_1_2_1_2_1_	P4_1_2_1_2
Unit cell dimensions		
*a*, *b*, *c* (Å)	60.25, 97.26, 146.73	100.57, 100.57, 147.83
*α*, *β*, *γ* (˚)	90.0, 90.0, 90.0	90.0, 90.0, 90.0
No. of unique reflections	71,290 (3483)	34,389 (3274)
Completeness (%)	99.5 (99.8)	99.8 (98.6)
Multiplicity	4.6 (4.3)	7.8 (8)
*R*_merge_ (%)^b^	6.2 (69.4)	7.4 (53.3)
CC1/2	0.99 (0.803)	0.98 (0.925)
<<I>/σ(<I>)>^c^	13.5 (2.0)	11.7 (2.9)
Refinement		
Resolution (Å)	81.07–1.87	59.63–2.30
*R*_work_ (%)^d^	17.92	19.35
*R*_free_ (%)^e^	22.24	24.47
Wilson *B*-factor (Å^2^)	33.637	55.09
rmsd		
Bond length (Å)	0.009	0.008
Bond angles (˚)	1.461	1.423
Ramachandran plot		
Favored (%)	99	98
Allowed (%)	1	2
Outliers (%)	0	0

a Numbers in parentheses correspond to the highest resolution shell.

b *R*_merge_ = Σ|*I*–(I)|/Σ*I*, where *I* is the integrated intensity of a given reflection.

c *</σ(<I>)> =* mean *I*_h_ over the standard deviation of the mean *I*_h_ averaged over all reflections in a resolution shell.

d *R*_work_ = Σ||*F*_o_| − |*F*_c_||/Σ|*F*_o_|, where |*F*_o_| is the observed structure factor amplitude and |*F*_c_| is the calculated structure factor amplitude.

e *R*_free_: *R*_factor_ based on 5% of the data excluded from refinement.

The overall structure of Ms_orn consists of the typical RNase-H fold of DnaQ-like exoribonuclease superfamily of proteins consisting of five central β sheets, β5-β4-β1-β2-β3 (arranged in ↑↑↑↓↑ direction) and nine α helices, along with an additional C-terminal helix, α10 (Fig. 3, *B* and *C*). The final model of Ms_orn in each subunit could be traced from residues 1 to 196 of the full-length protein. Although the terminal residues 197 to 209 of Ms_orn lacked any discernible density, a significant part of the unique C-terminal tail of Ms_orn (residues 181–196) could be built and formed the additional helix, α10, through residues 186 to 195. α10 of one subunit packs against the other subunit of the dimer in a “handshake”-like manner, creating a small flap over the other protomer (Fig. 3*B*) and is unique to Ms_orn among all bacterial Orn structures (Fig. 3*D*). Average *B*-factors of the C-terminal tail (residues 181–196) were found to be slightly higher than the rest of the chain (41.58 Å^2^ or 43.56 Å^2^ for the tail in the two subunits *versus* 33.59 Å^2^ or 36.91 Å^2^ for the N-terminal region, *i.e.*, residues 1–180), indicating slightly larger flexibilities for the tail region in both subunits.

### Role of C-terminal helix, α10, at the dimeric interface

In order to obtain better understanding of the additional C-terminal helix, α10, in Ms_orn, the structure of Orn of *Escherichia coli* (Eco_orn) with the canonical Rnase-H fold was also determined for comparative structural analysis. The final structure of Eco_orn could be traced from residues 2 to 181 of the polypeptide chain. Eco_orn was also identified as a dimer by gel filtration, SEC–MALLS and PISA (Fig. S1). The structure refinement statistics for the final model are given in Table 1. Superposition of Eco_orn structure over Ms_orn indicates overall structural similarity with rmsd of 1.134 Å across 347 C^α^-atoms of the dimer but lacking the C-terminal tail of Ms_orn (Fig. S1).

**Figure 4:**
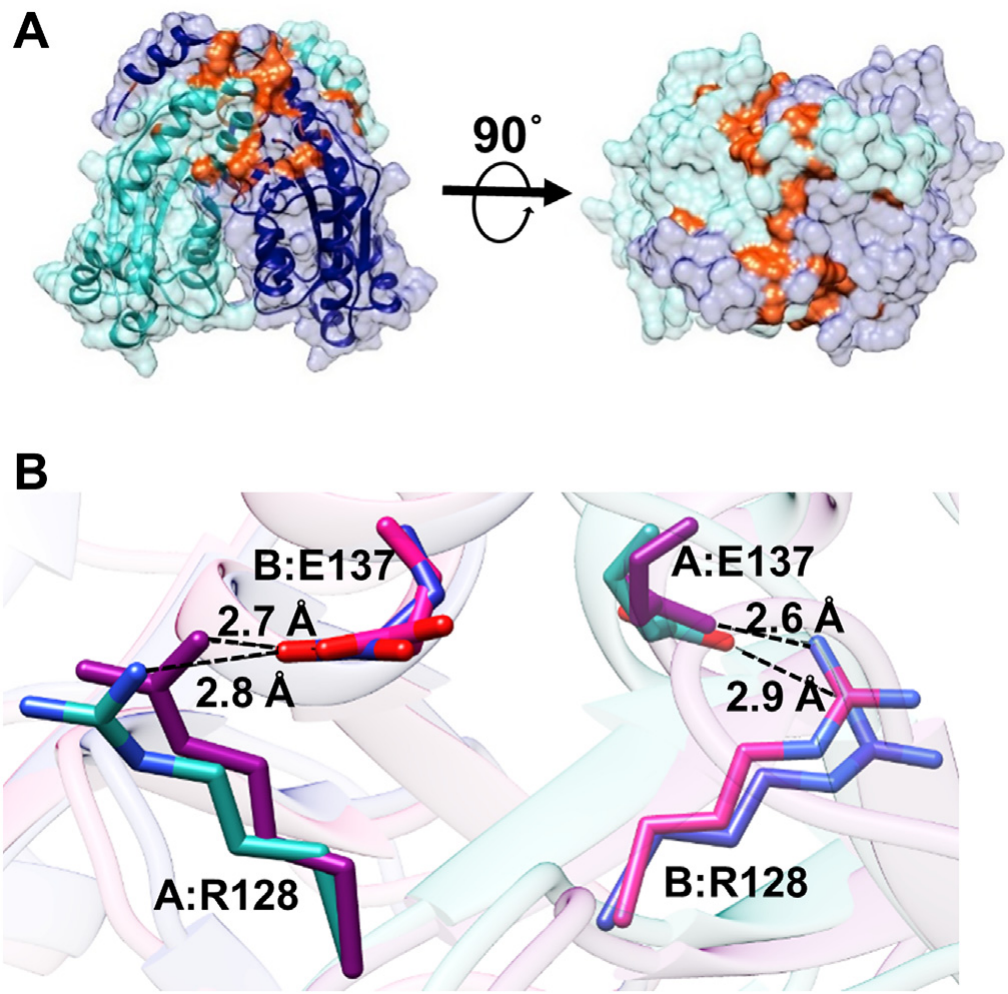
**Dimer interface of Ms_orn**. *A*, *front and top views* of the surface of Ms_orn with the two subunits shown in *sea green* and *navy blue colors*, whereas the interface is highlighted in *light red color*. *B*, the two salt bridges identified at the dimer interface in Ms_orn dimer (*cyan* and *navy blue*) and Eco_orn (*pink* and *magenta*) are shown. Residue labels corresponding only to Ms_orn are shown for clarity. Eco_orn, Orn of *Escherichia coli*; Ms_orn, Orn of *Mycobacterium smegmatis*.

As the C-terminal helix, α10, of Ms_orn packs against the opposite protomer and appears to aid the dimeric packing of the two subunits, the structures of Ms_orn and Eco_orn were first analyzed for differences in the dimer interface through PISA (45). Ms_orn buries 22.2% of total solvent-accessible area comprising nearly 2550 Å^2^ in the dimer interface (Fig. 4*A*). The dimer interface of Ms_orn is stabilized by two salt bridges (B: Arg128 [NH1]–A: Glu137 [OE2] and B: Glu137 [OE2]–A: Arg128 [NH1]) (Fig. 4*B*) and an additional 25 hydrogen bonds (Table S2).

Eco_orn, on the other hand, buries only 17.2% of total solvent-accessible area comprising nearly 1650 Å^2^ in the dimer interface. The residues involved in dimer formation in Ms_orn are also conserved in Eco_orn, with the dimer interface in Eco_orn being stabilized by the conserved salt bridges between B: Arg130 [NH1]–A: Glu139 [OE2] and B: Glu139 [OE2]–A: Arg130 [NH1]) (Fig. 4*B*) and an additional set of 16 H-bonds. The nature and position of residues forming H-bonds at the interface is similar in both proteins (Table S2). Solvent-accessible surface area of Ms_orn dimer interface, without the C-terminal helix α10 (residues 181–198), would be similar to that of Eco_orn, that is, 1580 Å^2^ and stabilized by the two conserved salt bridges and an equivalent number of H-bonds (18 predicted H-bonds without the C-terminal helix). The C-terminal helix hence provides additional interactions for dimer formation by burying an additional ∼1000 Å^2^ surface area in Ms_orn. A close-up view of interactions of the C-terminal tail of one chain with the second protomer indicates that hydrophobic and van der Waal interactions are the predominant interactions that help in packing of the subunits in this region (Figs. 3*B* and S2). Only the side chain of Gln181 of the C-terminal tail takes part in H-bond interactions (with main chain O of Trp142). Additional H-bonds at the interface involve main chain atoms of the C-terminal tail only (Gly183 [O]–Arg145 [NH2], Pro184 [O]–Arg145 [NH2], Table S2). As the side chains of residues 181 to 196 in the C-terminal helix are primarily short aliphatic/hydrophobic in nature (Figs. 2 and 3), an additive effect of weak van der Waal interactions appears to play a role in packing the two subunits.

### Interactions with modeled pGpG in the active site

**Figure 5:**
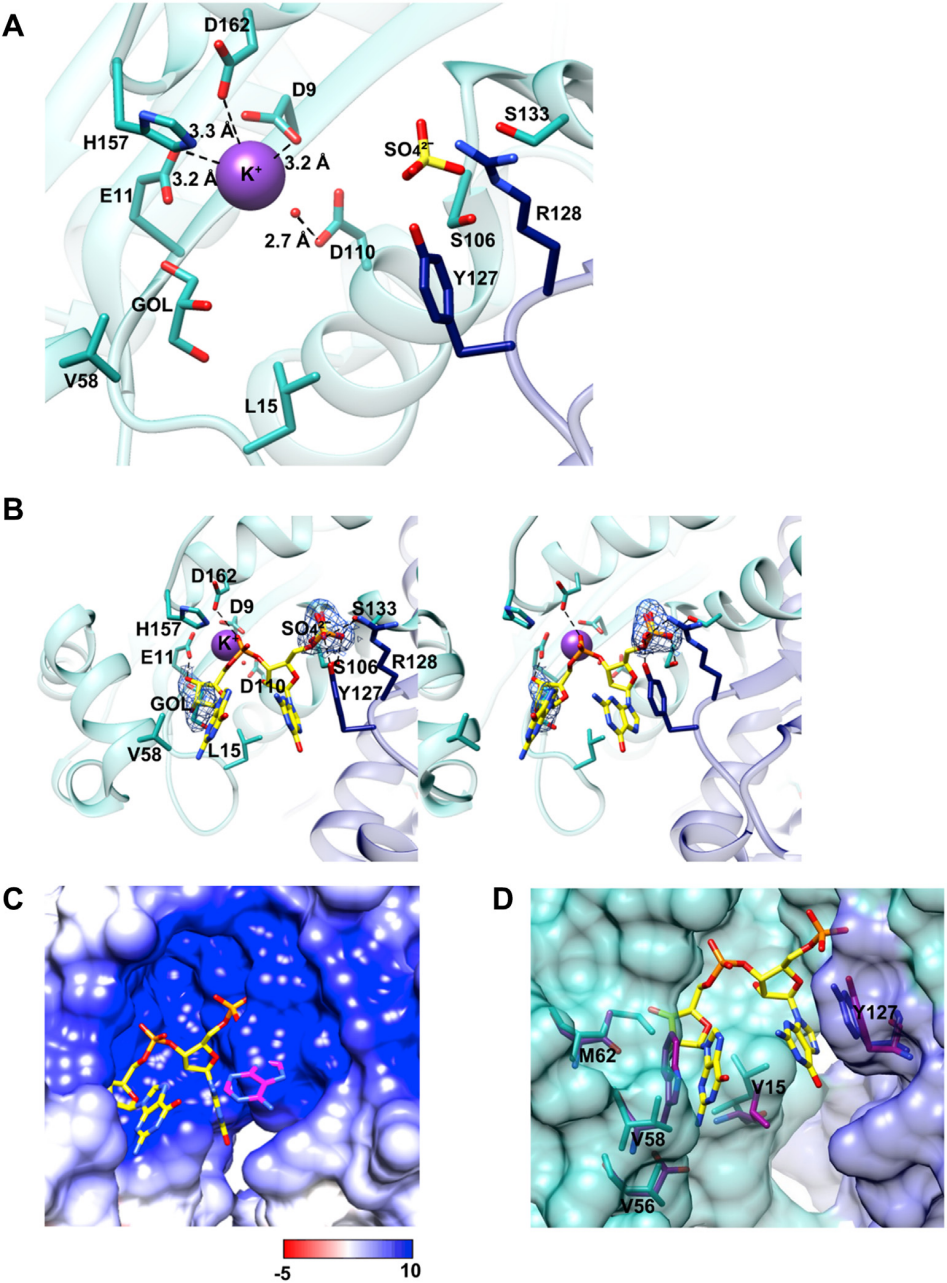
**Active site of Ms_orn and interactions with modeled substrate**. *A*, active site of Ms_orn showing conserved catalytic DEDDh residues coordinated to a metal ion. *B*, stereo image of active site of Ms_orn with a modeled pGpG substrate. 5′ Phosphate and 3′OH of modeled pGpG are occupied by a sulphate and glycerol molecule, respectively, at equivalent positions. A 2*F*_o_–*F*_c_ map for the glycerol and sulphate molecules of Ms_orn is also shown, contoured at 1.5 sigma level. *C*, the electrostatic surface potential of Ms_orn indicates a highly positive charged surface on the active site face of the protein (*red*: −5 kBT/e; *blue*: 10 kBT/e). A modeled 3-mer ribonucleotide shows steric clashes for the additional -1 nucleotide (*magenta*). The other two nucleotides (corresponding to pGpG) are shown in *yellow*. *D*, view of binding pocket showing the hydrophobic nature of catalytic pocket of Ms_orn is conserved through the presence of Val56, Val58, and Met62 replacing Asp59, Trp61, and Thr65. Ms_orn and Eco_orn residues are shown in *cyan* and *magenta*, respectively. A pGpG molecule (*yellow*) is also shown. Eco_orn, Orn of *Escherichia coli*; Ms_orn, Orn of *Mycobacterium smegmatis*.

The active site of Ms_orn and additional structural features in the substrate-binding pocket were next analyzed. The catalytic Asp9, Glu11, Asp110, and Asp162 along with the general base His157, which are characteristic of DEDDh family of exoribonucleases, are conserved in the active site of Ms_orn, as expected (Fig. 5*A*). In the absence of a bound substrate in the apo Ms_orn structure, His157 of Ms_orn was found to be disordered in one of the subunits. Interestingly, this active site histidine is present in a flexible loop and was observed to be disordered in several other apo Orn structures (46, 47), including Eco_orn. The carboxylate side chains in the active site of Orns usually coordinate two divalent metal ions for substrate binding and catalysis. In the apo form of Ms_orn, we observed only one monovalent cation, K^+^ ion, that was coordinated by Asp9 and Glu11 in all the chains (Fig. 5*A*).

In order to understand the structural basis of substrate binding in Ms_orn, pGpG was modeled in the active site by superposing the structure of *V. cholerae* Orn (Vc_orn)–pGpG complex (PDB ID: 6N6A) (46). Vc_orn superposes very well with Ms_orn with an rmsd of 0.908 Å for 348 aligned residues allowing the placement of pGpG into the Ms_orn active site and providing a template for further analysis of interactions of pGpG with active site residues of Ms_orn. The 5′ phosphate group of modeled pGpG was found to overlay a bound sulphate ion of Ms_orn, whereas the 3' OH of modeled pGpG overlays a bound glycerol molecule, suggesting equivalent binding site for the substrate in the two structural homologs (Fig. 5*B*). The 5' phosphate of modeled pGpG interacts with Ser106 and Ser133 of one subunit and Tyr127 and Arg128 of the other subunit and constricts the active site of Ms_orn (Fig. 5*B*). A similar set of residues has earlier been reported to cap the 5' end of pGpG substrate in Vc_orn, thereby constricting the active site and preventing binding of substrates longer than diribonucleotides and was termed as a “phosphate-cap” or P-cap (46). We examined the Orn sequences and found that the P-cap residues are conserved among them (Fig. 2) and hence may be a common structural feature among all Orns.

Fitting of a longer oligonucleotide substrate, for example, a 3-mer in Ms_orn active site showed steric clashes of the additional nucleotide at the -1 position, with P-cap residues, Tyr127 and Arg128 (Fig. 5*C*). The catalytic pocket of Ms_orn is also lined by Glu11, His63, Val59, and Met12, which prevent accommodation of oligonucleotides that are longer on the 3' end of pGpG, as suggested earlier (47). Notably, Glu11, Met12 and His63 are conserved among Orn sequences (Fig. 2), suggesting conservation of this “3′-OH capping” mechanism as well in the bacterial enzymes and limiting the binding site access to a 2-mer substrate only.

Additional interactions with the bound pGpG substrate in Vc_orn are through stacking interactions of the 5' and 3' nucleotide bases with a Tyr and Trp residue, respectively, with a conserved Leu forming a wedge between the two bases (46, 47, 48). In Ms_orn, Tyr127 and Leu15 were identified with the respective roles at equivalent positions although the Trp is replaced by Val58. Although the presence of a nonpolar aliphatic group in place of the bulky Trp slightly increases the pocket size, the hydrophobicity of the substrate-binding pocket is maintained by additional compensatory Val56 and Met62 substitutions in Ms_orn replacing Asp59 or Thr65 of Eco_orn, respectively (Fig. 5*D*).

The overall structural features of Ms_orn, residues at the active site and potential interactions with substrates hence appear to be highly conserved. The primary difference identified in the structure is the additional C-terminal helix, α10, that provides additional interactions at the dimer interface and appears to have no direct role in substrate binding. The role of this C-terminal helix in activity and/or stability was next investigated.

### Role of C-terminal tail in protein stability

Any possible role of the extended C-terminal tail on stability was investigated by comparison of thermodynamic parameters of Ms_orn and its deletion mutant (lacking the C-terminal tail) to thermal and chemical (guanidium hydrochloride [Gdm-HCl])–induced denaturation and measuring changes in their secondary structure. First, a C-terminal deletion mutant of Ms_orn, lacking residues 181 to 209, was constructed and termed as Ms_ornΔC'. However, Ms_ornΔC' could not be obtained in soluble form. Ms_ornΔC (lacking residues 186–209) was hence alternately expressed and could be obtained in soluble form as a dimer (Fig. S3) for further experiments.

**Figure 6:**
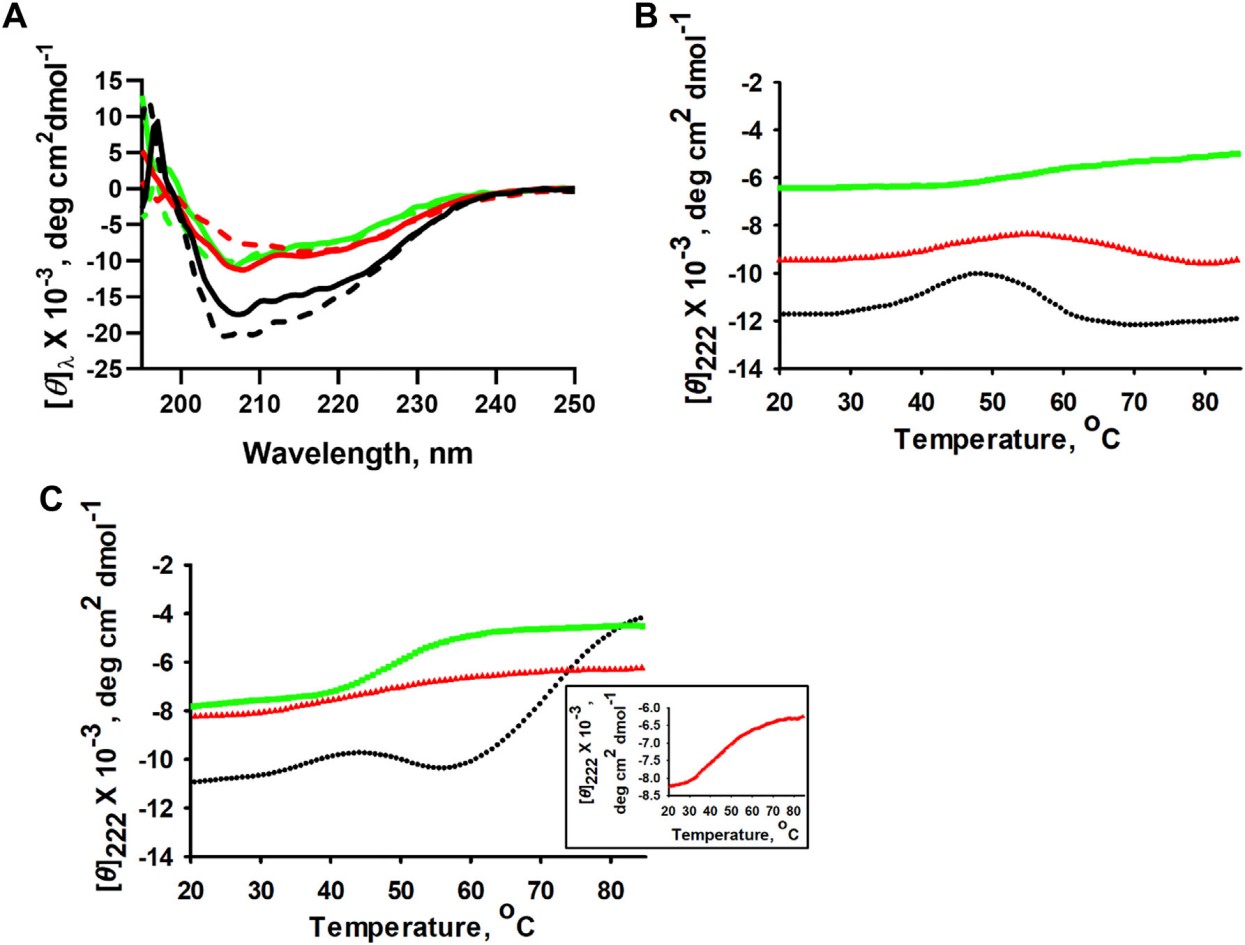
**Thermal denaturation profile of Ms_orn**. *A*, far-UV CD spectra of Ms_orn (*black*), Ms_ornΔC (*red*), and Eco_orn (*green*). Spectra were measured at 25 °C for the proteins under native buffer conditions (*solid lines*) and once again after prior heating at 85 °C for 10 min for monitoring reversibility (*dashed lines*). *B*, thermal denaturation in native state was monitored by following changes in [*θ*]_222_ nm, as a function of temperature. *C*, thermal denaturation profile in the presence of 0.3 M Gdm-HCl. *Inset* shows enlarged view of Ms_ornΔC denaturation profile. All thermal denaturation experiments and CD spectra measurements were carried out in triplicate. Eco_orn, Orn of *Escherichia coli*; Gdm-HCl, guanidium hydrochloride; Ms_orn, Orn of *Mycobacterium smegmatis*.

The secondary structures of Ms_orn, Ms_ornΔC and Eco_orn were first analyzed using far-UV CD spectra measurements in wavelength range of 195 to 250 nm at 25 °C. Figure 6*A* shows characteristic spectra of α/β-type proteins with negative peaks at 208 and 225 nm. Although both Ms_orn and Eco_orn are dimers and have similar structural content, Ms_orn has a slightly more negative far-UV CD signal than Eco_orn. Interestingly, the far-UV CD signal for Ms_ornΔC is similar to that of Eco_orn, possibly because of loss of the additional helical signal of the C-terminal helix, α10.

The Orns were then subjected to heat in the temperature range of 20 to 85 °C to monitor their thermal denaturation profiles. No change in [*θ*]_222_ nm in any of the proteins was observed as a function of temperature although Ms_orn shows a slight change in [*θ*]_222_ nm between 40 °C and 60 °C (Fig. 6*B*). There is a heat-induced increase in [*θ*]_222_ nm up to 50 °C, possibly because of partial unfolding but again appears to retain a state that exhibits [*θ*]_222_ nm signals similar to the pretransition state (Fig. 6*B*). Reversibility of the spectra was monitored by measuring [*θ*]_λ_ nm of all three Orns at 20 °C after first heating the proteins to 85 °C. The spectra were fully reversible and trace the spectra as obtained under native buffer conditions (Fig. 6*A*), suggesting high conformational stability for the orn dimers.

Since the proteins appear to be thermostable under the aforementioned conditions, we next performed thermal denaturation experiments in the presence of small amount of a denaturant to enhance the unfolding of proteins. For Eco_orn, a sharp transition in temperature range 42 to 60 °C was observed, which is indicative of a major conformational change suggesting unfolding of the protein with an estimated *T*_m_ of 50 °C. Thermal unfolding of Ms_ornΔC was also observed with 0.3 M Gdm-HCl, and the *T*_m_ estimated to be 50 °C with no intermediate transitions at lower temperatures.

In case of Ms_orn, however, again an intermediate transition from 36 to 55 °C similar to that of its thermal denaturation curve in the absence of Gdm-HCl was observed, followed by the major unfolding transition with an estimated *T*_m_ of 70 °C (Fig. 6*C*). The change in secondary structure content of Ms_orn at the intermediate transitions was estimated by K_2_D_2_ program of Dichroweb server. Under the native buffer conditions, a small change in alpha-helical content between 40–50 °C (corresponding to the observed intermediate transition of 36–55 °C) was observed and the helical content is restored at temperatures >55 °C to that observed at 25 °C (Fig. S4). Similarly, helical content of Ms_orn (with 0.3 M Gdm-HCl) is also first marginally reduced suggesting a transient local unfolding between 35 and 45 °C, followed by a slight increase in helical content at 50 °C before unfolding completely. In contrast to thermal unfolding under native conditions, thermal denaturation in the presence of 0.3 M Gdm-HCl (but not in its absence) was found to be irreversible (Figs. 6*C* and S2), suggesting that the low amount of denaturant was sufficient to perturb local structure to enable unfolding.

### Gdm-HCl induced denaturation of Ms_orn and Eco_orn

Temperature-induced conformational changes confer that Ms_orn is capable of tolerating higher temperatures than the C-terminal deletion mutant. To further investigate the conformational stability and denaturant-dependent effect on stability calculations, equilibrium Gdm-HCl–induced denaturation measurements were done by far-UV CD and intrinsic fluorescence measurements and thermodynamic parameters were calculated.

**Figure 7:**
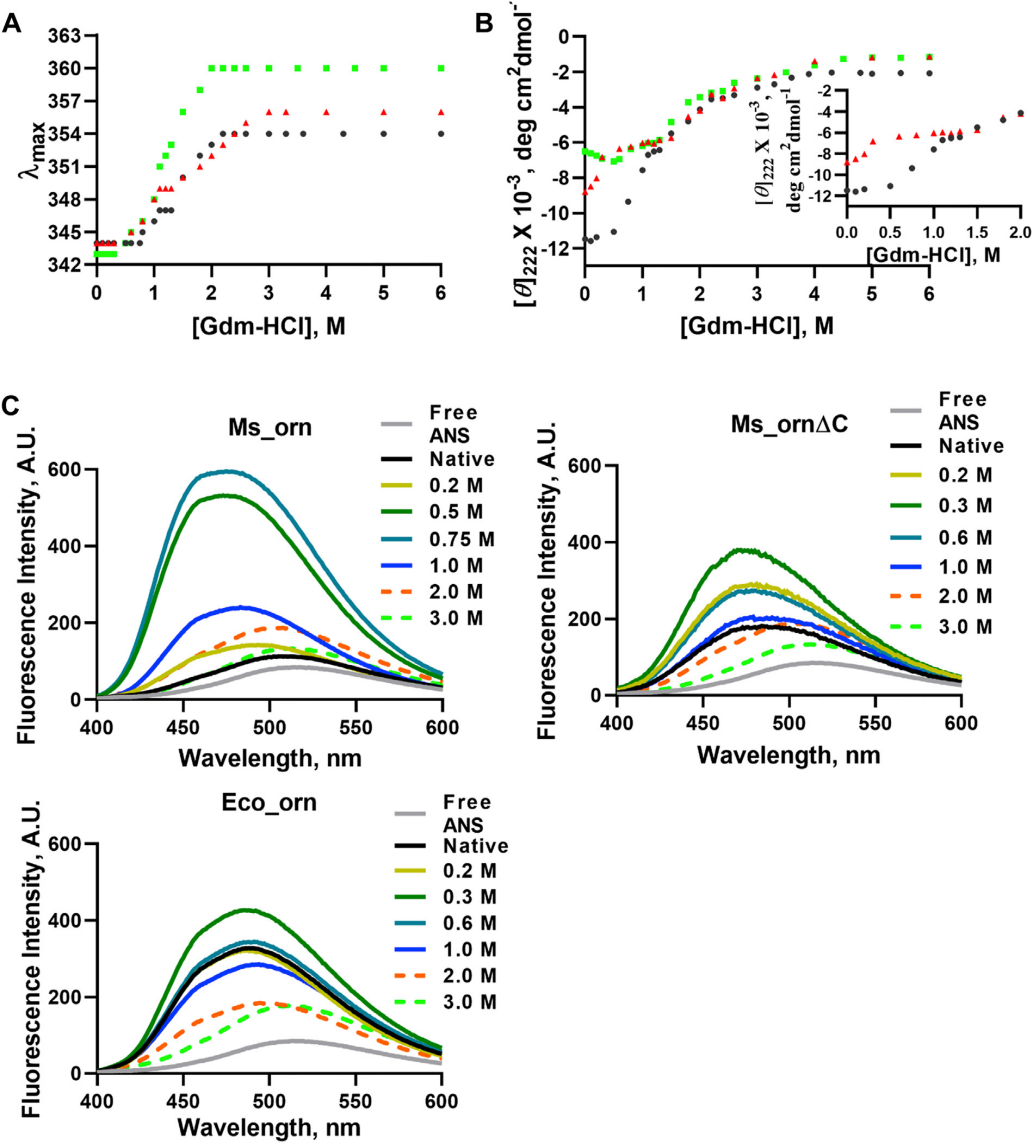
**Gdm-HCl–induced denaturation**. Gdm-HCl–induced denaturation of Ms_orn (*black*), Ms_ornΔC (*red*), and Eco_orn (*green*) monitored by following changes in (*A*) λ_max_ and (*B*) [*θ*]_222_ nm. *Inset* shows enlarged view for Ms_orn and Ms_ornΔC in the range of 0 to 2.0 M Gdm-HCl. *C*, ANS binding measurements for Ms_orn, Ms_ornΔC, and Eco_orn with increasing concentrations of Gdm-HCl, as indicated. The biphasic transition was highly reproducible. The unfolding curve with indicated set of of Gdm-HCl concentrations (*i.e.*, 0–6 M), used for estimation of parameters, was repeated twice before plotting. ANS, 8-anilino-1-naphthalene sulfonic acid; Eco_orn, Orn of *Escherichia coli*; Gdm-HCl, guanidium hydrochloride; Ms_orn, Orn of *Mycobacterium smegmatis.*

To monitor Gdm-HCl–induced denaturation of Ms_orn, Ms_ornΔC, and Eco_orn, intrinsic fluorescence spectra were measured at 25 °C in the presence of increasing concentrations of Gdm-HCl. The λ_max_ of fluorescence in the unfolded state reaches 356 nm for Ms_orn, 354 nm for Ms_ornΔC and 360 nm for Eco_orn. The denaturation curve of Eco_orn shows a monophasic manner of transition from folded to unfolded state, whereas both mycobacterial proteins exhibit a biphasic transition. Ms_orn denaturation profile shows a pre-transition state from 0 to 0.75 M Gdm-HCl concentration and intermediate transition shows stable conformation at 1.1 to 1.3 M Gdm-HCl followed by a completely unfolded form at 3.0 M Gdm-HCl (Fig. 7*A*). A size-exclusion profile of Ms_orn with increasing concentrations of Gdm-HCl over a Superdex-75 column revealed the presence of a dimer up to 1.0 M concentration, suggesting that the first transition is more likely to be local conformational changes (rather than a monomer–dimer transition) followed by unfolding (Fig. S5). A similar kind of nearly identical biphasic transition curve is observed for Ms_ornΔC with its pre-transition state from 0 to 0.6 M Gdm-HCl, intermediate transition from 1.1 to 1.3 M Gdm-HCl followed by a completely unfolded form at 3.0 M Gdm-HCl.

Gdm-HCl–induced denaturation was also monitored through far-UV CD, by measuring the change in [*θ*]_222_ nm as a function of increasing molar concentrations of Gdm-HCl (Fig. 7*B*). Ms_orn and Ms_ornΔC once again show a biphasic denaturation curve, as seen in their respective fluorescence spectra. The change in secondary structure of Eco_orn also follows a biphasic transition when monitored by [*θ*]_222_ nm although not by tryptophan fluorescence. The biphasic transitions as monitored by change in λ_max_ or [*θ*]_222_ are subtle yet highly reproducible in several experimental runs with varying concentrations of GdmCl between 0 to 3 and 4 M. The unfolding curve with indicated set of Gdm-HCl concentrations (*i.e.*, 0–6 M) was finally plotted as shown in Figure 7, *A* and *B* for estimation of thermodynamic parameters. The thermodynamic parameters for unfolding of Ms_orn, Ms_ornΔC and Eco_orn, as monitored by [*θ*]_222_ nm, were estimated for both transitions, that is, transition I (N to X) and transition II (X to U), where N represents native state, X is thermodynamically stable intermediate and U is unfolded state (Table 2 and Fig. S6). There is a major difference in [*θ*]_222_ nm of Ms_ornΔC in the 0 to 0.6 M Gdm-HCl range, which corresponds to transition I, that is, N ↔ X with much lower values of *C*_mI_ and Δ*G*_I_. In addition, the overall Δ*G* value of denaturation (Δ*G*_I_^0^ + Δ*G*_II_^X^) for Ms_ornΔC is also lower than that observed for Ms_orn (or Eco_orn) (Table 2), implying much lower stability of the C-terminal deletion mutant.

**Table 2:** Thermodynamic parameters associated with Gdm-HCl–induced denaturation of Ms_orn, Ms_ornΔC, and Eco_orn

Transition	Thermodynamic parameter	Ms_orn	Ms_ornΔC	Eco_orn
N ↔ X	Δ*G*_I_^0^	3.9 ± 0.03	1.94 ± 0.06	3.7 ± 0.17
	*C*_mI_	0.86 ± 0.0	0.24 ± 0.01	0.58 ± 0.01
X ↔ U	Δ*G*_II_^X^	1.6 ± 0.06	1.5 ± 0.14	1.3 ± 0.02
	*C*_mII_	2.1 ± 0.02	1.98 ± 0.01	1.75 ± 0.0

Δ*G*_II_^X^, Gibbs free energy change associated with transition II where X state exists.

Units of Δ*G*_I_^0^ or Δ*G*_II_^X^ is kilocalorie mole^−1^, that of *<I > C*_mI_ or *C*_mII_ is M.

“±” represents the mean error from duplicate measurements.

To monitor conformational state of the intermediate in the first transition, 8-anilino-1-naphthalene sulfonic acid (ANS)–binding measurements of Ms_orn, Ms_ornΔC and Eco_orn were carried out with increasing Gdm-HCl concentrations. ANS fluorescence intensity of Ms_orn with low Gdm-HCl concentrations was approximately six times at 0.5 and 0.75 M and nearly twice at 1.0 M than its native (or fully denatured) state (Fig. 7*C*). Similarly, ANS fluorescence intensity for Ms_ornΔC was 1.5-2-fold higher at low Gdm-HCl concentrations of 0.2, 0.3 or 0.6 M than its native (or fully denatured) state (Fig. 7*C*). The increase in ANS fluorescence intensity for Ms_orn and Ms_ornΔC at low Gdm-HCl concentrations corresponds with their first-phase transitions and is commensurate with the presence of an intermediate state in their respective unfolding transitions (Fig. 7*B*). Quantum yield of ANS fluorescence for Eco_orn at its native state in the absence of Gdm-HCl, on the other hand, itself is two-fold higher than that of its fully denatured state, suggesting that native Eco_orn has partially exposed hydrophobic surfaces, whereas Ms_orn is more tightly packed. A marginal 1.3-fold increase in ANS fluorescence intensity is observed for Eco_orn at 0.3 M Gdm-HCl, but no corresponding intermediate state was obtained in Gdm-HCl–mediated unfolding experiments, confirming that the unfolding pathways of Ms_orn and Eco_orn are different.

### Molecular dynamics simulation studies to understand the role of C-terminal tail of Ms_orn

**Figure 8:**
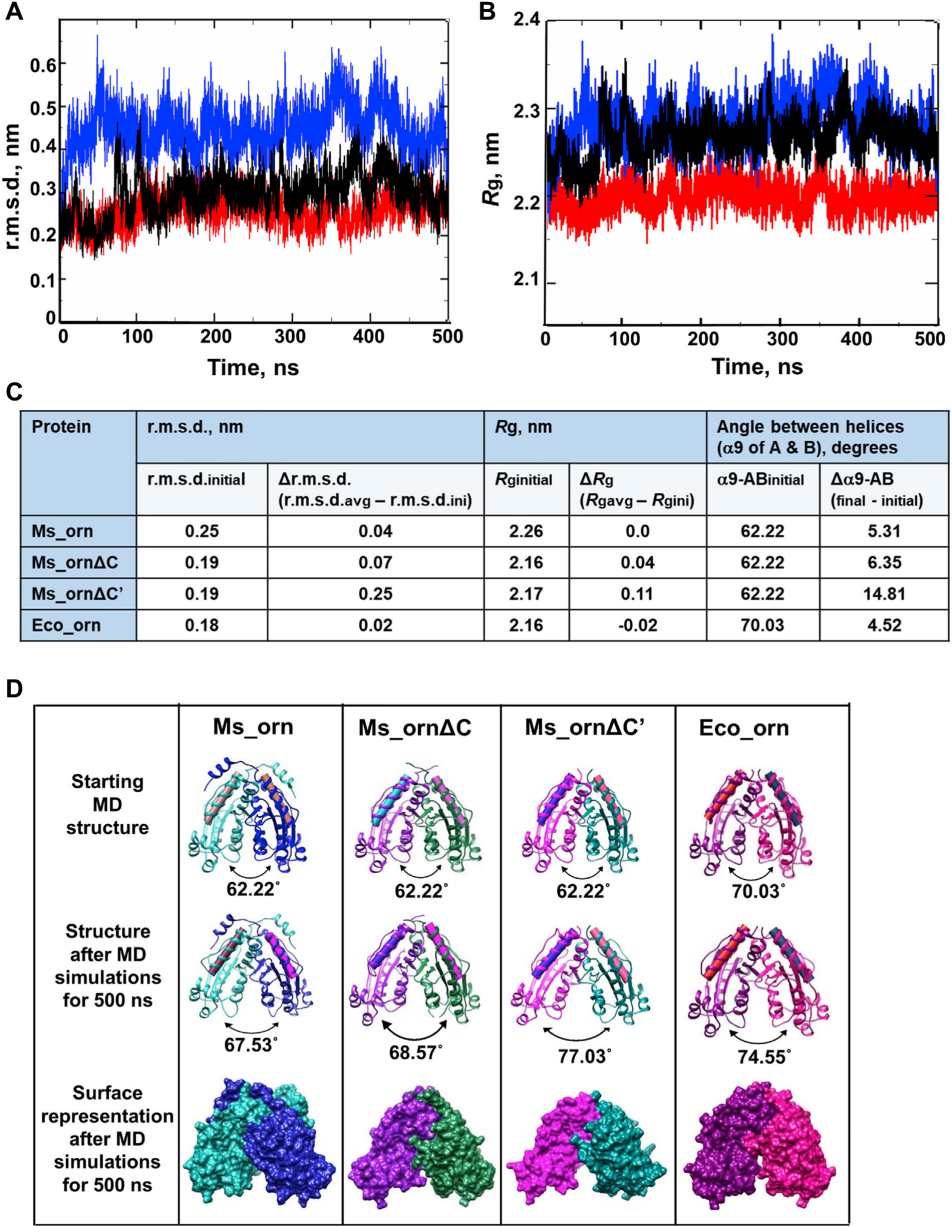
**MD simulation studies**. *A*, rmsd and (*B*) *R*_g_ plot of Ms_orn (*black*), Ms_ornΔC (*red*), and Ms_ornΔC’ (*blue*). *C*, measurement of changes in rmsd, *R*_g_, and opening of dimers (measured as angle deviation between helices α9 of the A and B subunits, shown as *cylinders*) indicate Ms_ornΔC′ has largest deviations. *D*, conformational analysis of Ms_orn, Ms_ornΔC, Ms_ornΔC′, and Eco_orn reveals opening up of dimers in all proteins after the MD simulations, with largest deviations in Ms_ornΔC’. Eco_orn, Eco_orn, Orn of *Escherichia coli*; MD, molecular dynamics; Ms_orn, Orn of *Mycobacterium smegmatis.*

Partial deletion of the C-terminal tail in the Ms_ornΔC mutant indicated subtle changes in stability of the protein, as estimated by thermal unfolding and Gdm-HCl–induced denaturation experiments. In order to assess the effect of deletion of the complete tail, we carried out an all-atom molecular dynamics (MD) simulations of Ms_orn, Ms_ornΔC, Ms_ornΔC′ and Eco_orn for 500 ns and analyzed C^α^-rmsd with respect to time. Our analysis indicated that with the deletion of the entire C-terminal tail of Ms_orn, the average rmsd (rmsd_avg_) values are higher in Ms_ornΔC' (0.44 nm) than that of native Ms_orn (0.29 nm) or Ms_ornΔC (0.26 nm), suggesting larger structural deviations in Ms_ornΔC′ during the MD run, than the other two mycobacterial proteins (Fig. 8*A*). The rmsd plot of Eco_orn also shows no major fluctuations during the timescale of MD simulations up to 500 ns (Fig. S7).

Compactness and structural changes during the course of MD simulations were also calculated through estimation of *R*_g_ as a function of time. Again, Eco_orn shows no major changes in *R*_g_ during the timescale of MD simulations up to 500 ns (Fig. S7). The mycobacterial proteins, Ms_orn and Ms_ornΔC, also show only a small deviation in *R*_g_ values at the end of the MD run, when compared with the initial values. Ms_ornΔC′, in contrast, exhibits the highest average *R*_g_ (*R*_g-avg_) = 2.26 nm and largest deviation of Δ*R*_g_ = 0.11 nm among the three mycobacterial Orn forms, despite estimated smaller size upon deletion of the C-terminal tail (Fig. 8, *B* and *C*).

A closer analysis of the simulated structures shows that there is a slight opening of the Orn dimer during the course of the MD run. While a marginal opening between 4.5º and 5.3º is observed in Ms_orn (or Eco_orn) dimers, Ms_ornΔC′ shows largest deviation of nearly 15º, corresponding with larger observed changes in *R*_g_ in this mutant (Fig. 8, *C* and *D*). A larger deviation in Ms_ornΔC′ when compared with Ms_ornΔC is possibly because of loss of all possible interactions at the C-terminal dimer interface in the former, whereas interacting residues, 181, 183 and 184, are retained in Ms_ornΔC.

### Effect of deletion of C-terminal tail of Ms_orn *in vivo*

**Figure 9:**
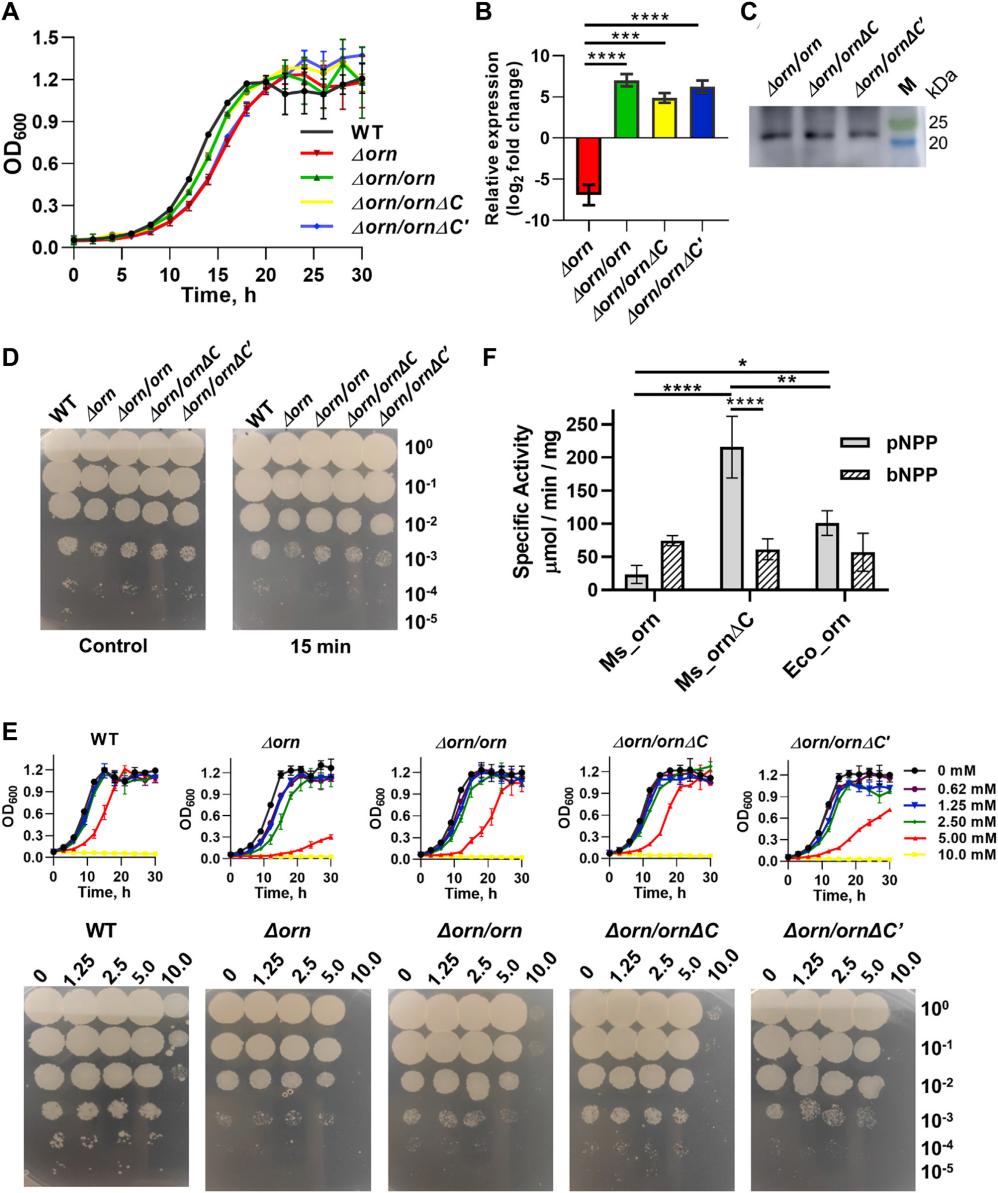
***In vivo* effect of deletion of C-terminal tail of Ms_orn**. *A*, growth curve of WT (*Mycobacterium smegmatis* mc^2^155, shown in *black*), *Δorn* (*red*), *Δorn/orn* (*green*), *Δorn/ornΔC* (*yellow*), and *Δorn/ornΔC’* (*blue*) in 7H9 media. *B*, relative expression of *orn* with respect to WT in *Δorn*, *Δorn/orn*, *Δorn/ornΔC*, and *Δorn/ornΔC’.* Average values of three independent biological replicates are plotted after normalization with the β′ subunit of RNA polymerase, *rpoC*; error bars indicate ± standard deviation. The *p* values were calculated by one-way ANOVA Tukey's post hoc test. *Asterisks* in figure represent significant difference between two groups (∗*p* < 0.05, ∗∗*p* < 0.01, ∗∗∗*p* < 0.001, and ∗∗∗∗*p* < 0.0001). *C*, expression of C-terminal deletion mutants in the soluble fractions was confirmed by Western blotting with a mouse anti-His antibody against full-length complement (orn/Δ*orn*) as a control. A prestained molecular weight marker is indicated (M). *D*, growth of indicated serial dilutions of WT, *Δorn*, *Δorn/orn*, *Δorn/ornΔC*, and *Δorn/ornΔC′* on 7H9 agar plates after exposure to UV for 15 min. *E*, *upper panels*, growth of WT, *Δorn*, *Δorn/orn*, *Δorn/ornΔC*, and *Δorn/ornΔC′* in the presence of increasing concentrations of H_2_O_2_ in 7H9 media with constant shaking. Growth of serial dilutions of WT, *Δorn*, *Δorn/orn*, *Δorn/ornΔC*, and *Δorn/ornΔC′* strains on 7H9 agar plate after incubation for 3 h with indicated concentrations of H_2_O_2_ (milimolar). All growth experiments were performed with two biological replicates. *F*, *in vitro* activity of Ms_orn. Phosphatase (*gray bars*) and phosphodiesterase (*slant lines*) activity of Ms_orn, Ms_ornΔC, and Eco_orn on pNPP and bNPP substrates, respectively. The *p* values of relative specific activity on pNPP were calculated by two-way ANOVA multiple comparison test (∗*p* < 0.05, ∗∗*p* < 0.01, ∗∗∗*p* < 0.001, and ∗∗∗∗*p* < 0.0001). The phosphatase (100.75 μmol/min/mg) and phosphodiesterase activity of Eco_orn (52.67 μmol/min/mg) on the same substrates is also shown for comparison. The error bars represent standard deviation of two independent experiments. bNPP, bis-(*p*-nitrophenol) phosphate; Eco_orn, Orn of *Escherichia coli*; Ms_orn, Orn of *Mycobacterium smegmatis*; pNPP, *p*-nitrophenol phosphate.

To establish the role of C-terminal tail in the activity of Ms_orn *in vivo*, a knockout of *orn* in *M. smegmatis* mc^2^155 (*Δorn*) (P.B. and B.T. [unpublished observations]) was complemented with clones expressing proteins corresponding to full length (*orn*), 1–185 residues (*ornΔC*) or 1–180 residues (*ornΔC′*). Growth of knockout and complemented strains was monitored by measuring optical density at 600 nm and compared with wild-type (WT) strain. Deletion of *orn* (*Δorn* strain) exhibited slightly slower growth as compared to WT, which was partially restored in the complemented *Δorn/orn* and *Δorn/ornΔC* strains but not in *Δorn/ornΔC′*, which lacks the complete C-terminal tail of Ms_orn (Fig. 9*A*). Expression of *orn* (or its mutant forms) in the soluble fractions was confirmed by monitoring transcript levels by quantitative RT–PCR (qRT–PCR) (Fig. 9*B*) and Western blotting (Fig. 9*C*), before comparing growth of WT, knockout and the complemented strains.

Optimal c-di-GMP levels help *M. smegmatis* modulate transcriptional network in response to environmental stress (49). The response of WT and mutant strains to environmental stress agents, was examined by exposure to UV (DNA damage) and varying levels of H_2_O_2_ (oxidative stress). Upon exposure to UV for 15 min, *Δorn/orn* and *Δorn/ornΔC* could restore growth up to 10^−4^ dilution, similar to WT, whereas growth in *Δorn/ornΔC′* remained similar to the knockout strain (Fig. 9*D*).

Upon exposure to varying levels of H_2_O_2_, no difference in growth was observed up to 2.5 mM H_2_O_2_ in the WT strain. Slightly reduced growth was observed at 5 mM and growth of the bacterium was completely inhibited at 10 mM H_2_O_2_. Growth was restored nearly to WT levels in both *Δorn/orn* and *Δorn/ornΔC* complemented strains and only marginally in the *Δorn/ornΔC′* complemented strain (Fig. 9*E*). Growth was also monitored by exposure to H_2_O_2_ for 3 h and spotting serial dilutions on 7H9 plates. *Δorn* and *Δorn/ornΔC′* showed no growth up to 48 h after exposure, suggesting that the presence of complete extended C-terminal tail is required for proper functioning of Ms_orn and to enable optimal growth of *M. smegmatis* under both stress and stress-free growth conditions.

The PDE activity of Ms_orn and Ms_ornΔC was also tested *in vitro* with bis-(*p*-nitrophenol) phosphate (bNPP) as a substrate that mimics the two rings of its *in vivo* pGpG substrate. Specific activity of Ms_orn on bNPP was found to be 74.36 μmol/min/mg. Deletion of the C-terminal tail in Ms_ornΔC leads to a slight decrease in PDE activity to 61.11 μmol/min/mg. The absence of the C-terminal helix, however, leads to more than nine-fold higher phosphatase activity in Ms_ornΔC than Ms_orn (Fig. 9*F*), suggesting that the C-terminal tail may play a role in substrate access and selection.

## Discussion

Homeostasis of c-di-GMP is key for normal physiology and survival of *M. smegmatis* as well as *M. tuberculosis.* C-di-GMP regulates lipid transport and metabolism (23), survival under hypoxic and reductive stress (21, 22, 49, 50), modulation of pathogenicity and antibiotic response in both mycobacterial species (20, 23, 24). The intracellular levels of c-di-GMP in bacteria are maintained by two antagonistic enzyme families: DGCs for its biosynthesis and PDEs containing either HD-GYP or EAL domain for its degradation (1, 3, 4) that hydrolyze c-di-GMP into GMP or pGpG, respectively. In mycobacteria, no homolog for HD-GYP domain containing PDE is present and c-di-GMP is hence cleaved to pGpG by EAL domain containing PDEs; the bifunctional DGC (MSMEG_2196) in *M. smegmatis* (21) and DGC (Rv1354c) or MtbPDE (Rv1357c) in *M. tuberculosis* (20, 51). Removal of pGpG is important to maintain the pool of free nucleotides inside the cell and to prevent mispriming of transcripts (41) and is brought about by Orns (33, 34). Any excess uncleaved pGpG can cause feedback inhibition on EAL domain containing PDEs and extend the half-life of c-di-GMP in the cell (4, 33, 34). Despite the importance of complete metabolism of c-di-GMP to GMP, when not required for its regulatory roles, the mycobacterial Orn remains uncharacterized so far. In addition, mycobacterial Orns have an extended C-terminal tail in contrast to other bacterial homologs. In order to obtain better insights into this enzyme, we have determined the crystal structure of MSMEG_4724, the Ms_orn, to investigate the role of its unique structural features and to examine the role of Ms_orn *in vivo*.

### The C-terminal tail adopts a helical conformation

The crystal structure of Ms_orn reveals that a significant part of the C-terminal tail forms an additional helix, α10, in addition to the core canonical RNase-H fold of Orns. This thumb-like helical protrusion of the C-terminal tail packs against the opposite protomer in a handshake-like manner of Ms_orn dimer (Fig. 3) and buries nearly 40% additional surface (∼1000 Å) in the dimer interface (Fig. 4*A*). Although the requirement of this additional packing at the dimer interface in Ms_orn but not in Eco_orn or other bacterial Orns is not evident, the role of the C-terminal tail in stabilization of the Ms_orn dimer was also demonstrated by larger deviations in MD simulations in Ms_ornΔC′, lacking the entire C-terminal tail but not in Ms_ornΔC (Fig. 8). Moreover, based on the predominant presence of residues with short/aliphatic side chains in the C-terminal helix of Ms_orn, we hypothesize that a cumulative additive effect of weak van der Waal forces comes into play in packing the two subunits.

Although the primary role of the C-terminal helix, α10, appears to be to aid packing of the Ms_orn dimer, the C-terminal tail is also required for activity of the protein. Growth of an orn knockout strain (*Δorn*) could be rescued by complementation by full-length Ms_orn (*Δorn*/*orn*) as well as a partial deletion of the tail (*Δorn/ornΔC*) but not by complete absence of the tail (*Δorn/ornΔC′*) (Fig. 9*A*). Deletion of *orn* also increases susceptibility of the *Δorn* strain toward stress agents like UV and oxidative stress (Fig. 9, *D* and *E*). Again, complementation by *ornΔC* but not by *ornΔC′* results in (partial) restoration of growth. Deletion of the (partial) C-terminal tail affects *in vitro* activity of Ms_orn on chemical substrates as well. The absence of α10 in Ms_ornΔC resulted in a nearly 10-fold increase in phosphatase activity on p-nitrophenol phosphate (pNPP) as a substrate (Fig. 9*F*). This large increase in phosphatase activity compared to only a small decrease in PDE activity (on bNPP) in Ms_ornΔC could be due to easier access to the active site for the smaller substrate (pNPP). The unavailability of Ms_ornΔC′ because of insolubility precluded estimation of effect of the complete deletion of the C-terminal tail on *in vitro* activity assays.

The “handshake” conformation of Ms_orn is hence a unique native conformation that aids packing of the dimer and also regulates activity through access of substrates into the active site in both *in vivo* and *in vitro* conditions.

### Ms_orn constrains a 2-mer substrate in its binding pocket though P-cap and 3′OH cap

We identified two bound ligands (a sulphate ion and a glycerol molecule) in the active site of Ms_orn that helped map molecular interactions of the protein with a potential substrate (Fig. 5*B*). In order to aid the structural basis of substrate binding in Ms_orn, pGpG was modeled in the active site using Vc_orn as a reference structure. Interestingly, the sulphate ion is present at the same site as the 5′ phosphate of the modeled pGpG and makes similar interactions with two serines of one subunit (Ser106 and Ser133) and a tyrosine and arginine of the other (Tyr127 and Arg128), as seen in Vc_orn. These residues have been defined as a 5′ P-cap that constricts the active site of Orns and prevent binding of substrates longer than diribonucleotides (46).

Modeling of a 3-mer oligo with an additional nucleotide at the 5' end of modeled pGpG showed steric clashes with Tyr127 and Arg128 of the P-cap (Fig. 5*C*). On the 3' OH side of the 2-mer, the binding pocket is lined by the catalytic Glu11 along with His63, Val59 and Met12 of Ms_orn (Fig. 5*D*). These residues prevent the fitting of a 3-mer oligo with an additional nucleotide at the 3' end of modeled pGpG. A similar observation was also made in REXO2 structure earlier (47). Indeed, examination of Orn sequences confirmed that residues corresponding to Glu11, Met12 and His63 of Ms_orn were conserved (Fig. 2) and we propose that they are likely to play a conserved “3' OH capping” role. The presence of both a conserved P-cap at 5' site and the proposed 3' OH cap on the 3′ site of modeled pGpG thereby limits the space in binding pocket for a 2-mer substrate only. Interestingly, crystal structures of Orns with bound oligos, all show only a 2-mer oligo in the binding pocket and additional nucleotides were found to be disordered or with very high *B*-factors, despite being used in crystallization trials (46, 48, 52).

Although a 2-mer has been shown to be a preferred substrate in both bacterial (*P. aeruginosa* and *V. cholerae*) and mammalian mitochondrial Orn (REXO2), recently (33, 46, 53), *in vitro* biochemical assays show that Orns bind and cleave longer ribonucleotides (typically 2–5 mer) as well (37, 38, 47, 54, 55). The active site face of Ms_orn is positively charged and additional nucleotides (longer than 2-mer) may be accommodated outside the binding pocket through nonspecific interactions on this side (Fig. 5*C*).

### C-terminal helix, α10, renders higher stability features to Ms_orn

Proteins of intracellular pathogens like *M. tuberculosis* have often been shown to have higher stability to retain their function *in vivo* (56, 57). We examined whether the C-terminal tail of Ms_orn could play such a role to enable higher stability to the protein by thermal and Gdm-HCl–induced denaturation experiments. First, thermal stability of Ms_orn and Ms_ornΔC was examined through [*θ*]_222_ nm. Both Ms_orn and Ms_ornΔC appear to be thermostable under our experimental conditions and no major change in [*θ*]_222_ nm was observed as a function of temperature for either protein. Thermal denaturation was hence next carried out in the presence of small amount of a denaturant to enhance the unfolding of proteins and yielded an estimated *T*_m_ of 70 °C for Ms_orn, which was much higher than that obtained for Ms_ornΔC (estimated *T*_m_ of 50 °C). Interestingly, estimated *T*_m_ (50 °C) of Eco_orn (which naturally lacks the C-terminal extended region) was similar to that of Ms_ornΔC, suggesting α10 aids the higher observed thermostability of Ms_orn (under mild denaturing conditions).

The structural stability of Ms_orn and role of the C-terminal tail were also examined by Gdm-HCl–induced denaturation experiments for Ms_orn and Ms_ornΔC. The denaturation profile of both Ms_orn and Ms_ornΔC exhibited a biphasic transition when monitored by either changes in λ_max_ of intrinsic fluorescence or [*θ*]_222_ nm (Fig. 7, *A* and *B*). The biphasic transition and the presence of the intermediate unfolding state for Ms_orn and Ms_ornΔC were confirmed by ANS binding experiments as well (Fig. 7*C*). SEC profile with low concentrations of Gdm-HCl (<1.0 M Gdm-HCl), (Fig. S5) shows the dimer is retained at these concentrations. The presence of hydrophobic regions (ANS binding) and apparent dimeric state (SEC with Gdm-HCl) suggests that Ms_orn unfolding is initiated by local perturbations first, followed by complete unfolding and loss of the dimeric state.

A lower *C*_mI_ of Gdm-HCl was required for first transition of Ms_ornΔC by both intrinsic fluorescence and CD measurements, possibly because of loss of additional dimer packing (that would have been provided by α10) in the full-length protein. Once again, Eco_orn, naturally lacking the C-terminal extended region, appears to follow a different unfolding pattern from Ms_orn when monitored by either changes in λ_max_ of intrinsic fluorescence or [*θ*]_222_ nm, suggesting that the unfolding pathways of Ms_orn with the extended C-terminal tail are different from other Orns.

**Figure 10:**
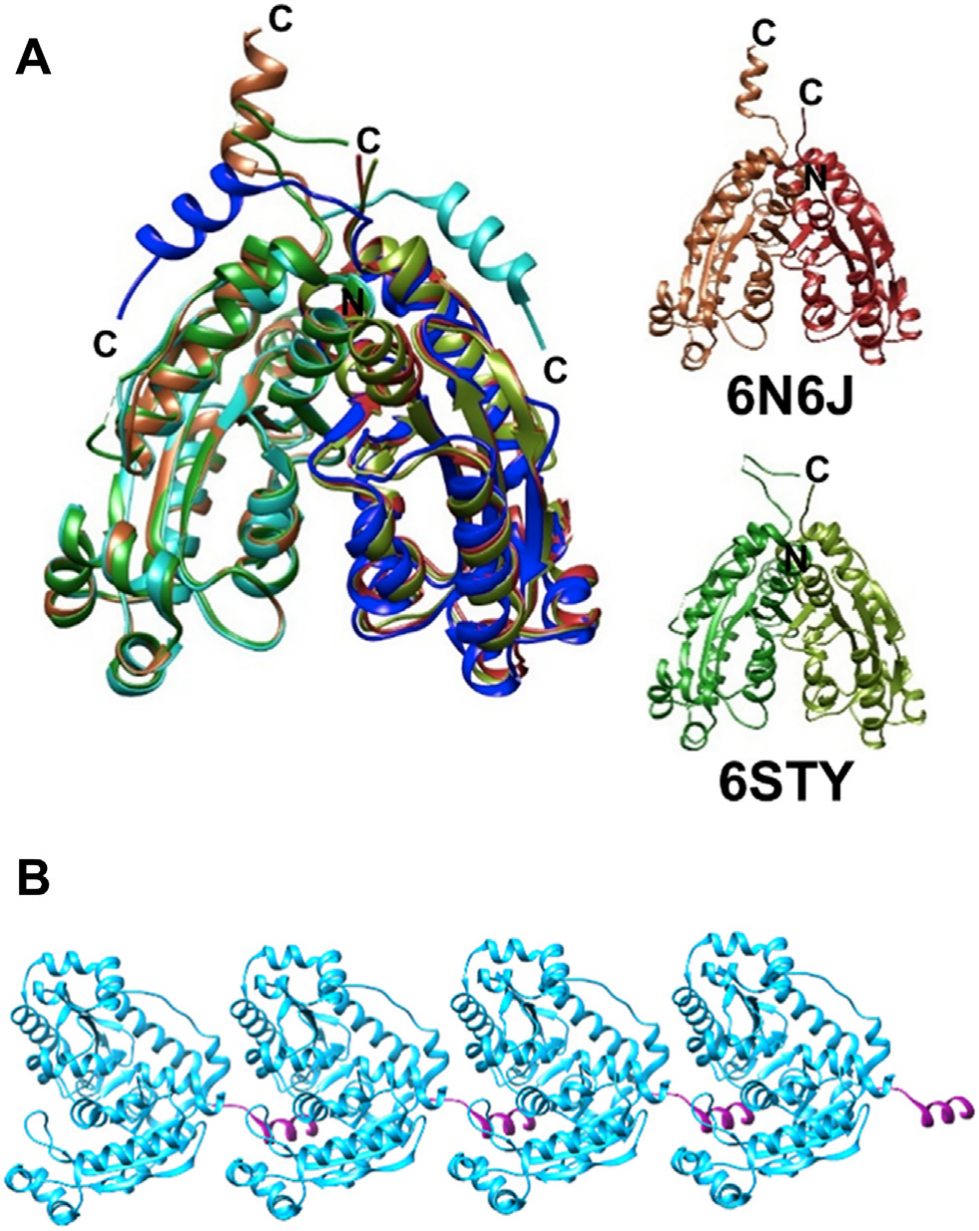
**C-terminal tail of REXO2.***A*, superposition of two REXO2 dimer structures, 6N6J (*brown*) and 6STY (*green*) over Ms_orn dimer (*cyan* and *blue*) to show the orientation of extended C termini. Ms_orn shares 48.8% sequence identity with both REXO2 structures. 6N6J and 6STY superpose very well with Ms_orn with rmsd of 0.969 and 1.109 Å for 352 and 348 aligned residues of the dimer, respectively. However, extended C-terminal helix is seen only in 6N6J but not in 6STY. *B*, crystal packing of REXO2 (6N6J) shows that the extended helix of REXO2 (*magenta*) is involved in crystal contacts. Ms_orn, Orn of *Mycobacterium smegmatis*.

The extended C-terminal tail of Ms_orn hence transmits several unique features to the protein. Examination of additional unique sequence features among RNase-H fold–containing proteins using DALI (58) indicated the presence of an additional C-terminal helix in one of the REXO2 (human mitochondrial Orn) crystal structures (PDB ID: 6N6J) (Fig. 10*A*). The C-terminal tail of REXO2, however, is involved in crystal contacts (Fig. 1*B*) and hence does not appear to be in its native conformation. Unfortunately, none of the other REXO2 structures exhibit this extended C-terminal region (and were presumed disordered), preventing further analysis of this region in REXO2. The C-terminal tail of Ms_orn is hence unique and likely to have organism-specific roles for maintaining the stability of this key enzyme for c-di-nucleotide homeostasis and its regulatory roles in growth and stress response in the harsh intracellular environments.

In conclusion, we present the structure of mycobacterial orn that helps in complete degradation and maintenance of c-di-GMP homeostasis in the cell. Ms_orn structure consists of the canonical RNase-H fold and contains an additional C-terminal helix that packs against the other protomer in a “handshake” conformation providing additional interactions at the dimer interface. We identify preference of a 2-mer substrate in Ms_orn through site constrictions in the substrate binding pocket by conserved residues constituting the P-cap or “3' OH cap.” Deletion of the C-terminal tail leads to opening up of the dimer as seen by MD simulation experiments, highlighting its importance. C-terminal tail is required for proper functioning of the enzyme as deletion of C-terminal tail of Ms_orn in bacterial cell affects its growth in both normal and stress conditions. Finally, Gdm-HCl–mediated unfolding and thermal denaturation experiments suggest that the C-terminal region helps in higher stability of Ms_orn, highlighting the role of intrinsic sequence features in mycobacterial sequences that enable its stable state under harsh intracellular conditions. This study sheds insights on the structure–function relationship of Ms_orn and its unique C-terminal region, which will be key in evaluating the intracellular levels and homeostasis of c-di-GMP in regulation of key pathways by this signaling molecule.

## Experimental procedures

### Chemicals and reagents

Sodium chloride, trizma, glycine, SDS, EDTA, ANS, Gdm-HCl, PMSF, and imidazole were obtained from Sigma Chemical Co. Nickel–nitrilotriacetate (Ni–NTA) agarose–based resin was brought from Qiagen. Mono-Q ion-exchange and superdex-75 (10/300 GL) gel filtration columns were purchased from GE Healthcare. LB agar, LB broth, glycerol, ammonium persulphate, IPTG, and kanamycin were purchased from Himedia Laboratories. Syringe filters (0.22 and 0.02 μm cutoff) were purchased from Millipore Corporation. All chemicals and reagents used were of analytical grade.

### Cloning, expression, and purification

Rv2511 is annotated as *M. tuberculosis* Orn (Mtb_orn). The open reading frame of *MSMEG_4724* (GenBank accession ID: CP000480.1), corresponding to a gene product of 216 amino acids, was identified as the ortholog of Mtb_orn and amplified from genomic DNA of *M. smegmatis* mc^2^155 using gene-specific forward and reverse primers (Table S1) to obtain two different clones. Clone A encodes full-length protein of 216 amino acid residues, whereas the second clone (clone B) encodes a product starting from eighth amino acid of MSMEG_4724, corresponding to N terminus of Mtb_orn. Both forward primers introduce a BamHI restriction site at 5′ end, and the reverse primer introduces a XhoI restriction site at 3′ end after the stop codon. The PCR amplicon was digested with BamHI and XhoI restriction enzymes and purified using gel-extraction kit. The digested product was cloned in pET-28-His_10_-Smt3 expression vector at BamHI and XhoI sites. The vector hence encodes clone A or clone B of MSMEG_4724 fused to His_10_-Smt3 (His_10_-tagged yeast SUMO protein, Smt3) tag at the N terminus. The recombinant construct was transformed into *E. coli* BL21 (DE3) for expression, and the tagged proteins were purified as described previously (59). In brief, recombinant protein was purified using Ni–NTA agarose beads, and His_10_-Smt3 tag was cleaved by Smt3-specific protease (Ulp1). Clone A of MSMEG_4724 yielded insoluble protein in inclusion bodies and could not be used further. The final purification steps to obtain purified protein from clone B required purification over an anion exchange column (Mono-Q; GE Healthcare) followed by SEC using Superdex-75 (10/300 GE) column (GE Healthcare) with 20 mM Tris–HCl, pH 8.5, and 100 mM NaCl as column buffer. Purity of the protein was checked on 12% SDS-PAGE. The purified MSMEG_4724 from clone B, thus obtained, was stored at −20 °C until further use and is referred to as Ms_orn in the rest of the article.

A C-terminal deletion mutant of Ms_orn, lacking residues 181 to 209 and annotated Ms_ornΔC', was constructed using specific forward and reverse primers (Table S1) to yield the truncated protein. However, Ms_ornΔC' was found in inclusion bodies and could not be purified in soluble form. Ms_ornΔC (lacking residues 186–209 at the C terminus of Ms_orn) was alternately expressed and purified following the same procedure as described previously for Ms_orn and used for all experiments reported for the C-terminal deletion mutant of Ms_orn.

The open reading frame corresponding to Orn (*orn*) of *E. coli* (National Center for Biotechnology Information accession ID: NC_000913.3) was amplified using PCR, from genomic DNA of *E. coli* K-12 strain, using gene-specific forward and reverse primers and cloned in pET-28-His_10_-Smt3 expression vector at BamHI and HindIII restriction sites. The protein, referred to as Eco_orn, was purified as described for Ms_orn and finally resuspended in 20 mM Tris–HCl, pH 8.5, and 100 mM NaCl and stored at −20 °C in 100 μl aliquots until further use.

### SEC–MALLS of purified protein

SEC with in-line MALLS (SEC–MALLS) was performed using ÄktaPure (GE Healthcare) FPLC connected in series to Dawn Heleos8+ multiangle laser scattering detector and Optilab T-rEX, a refractive index detector (Wyatt Technologies) (60). SEC was performed using Superdex-75 (10/300 GE) column at room temperature in 20 mM Tris–HCl, pH 8.5, 100 mM NaCl, and 5% glycerol, operated at 0.5 ml/min flow rate. About 200 μl of 3 mg/ml of Ms_orn or 6 mg/ml of Eco_orn were injected on the column in separate runs. ASTRA 7.3.1.9 software (Wyatt Technologies) was used to collect data from UV, refractive index and light scattering detectors for further analysis. Absolute molecular weight of protein (*M*_w_) was determined using *dn/dc* of 0.1850 ml/g for both Ms_orn and Eco_orn. To normalize light scattering signal across detectors, bovine serum albumin (Millipore Sigma) was used as a standard in the same buffer and at same flow rate. All measurements were carried out in duplicates.

### Crystallization, data collection, and structure refinement

Ms_orn was crystallized by hanging drop diffusion method by mixing 2 μl of 0.3 mM protein in 20 mM Tris–HCl buffer, pH 8.5, 100 mM NaCl, and 2 mM MgCl_2_ and 2 μl of reservoir solution (0.1 M Tris–HCl buffer, pH 8.4, 0.2 M lithium sulphate, and 30% v/v PEG-4000) at 24 °C. Long and thick rod–shaped crystals were obtained after 8 to 10 days and flash frozen in liquid nitrogen in cryo-solution (reservoir solution containing 10% glycerol) before data collection.

Diffraction data were collected remotely at ID29 beamline at European Synchrotron Radiation Facility. Data were processed and integrated using autoPROC 1.1.7 (20171219) (61) and scaled using XSCALE (62). Statistics for data collection and refinement are summarized in Table 1.

Ms_orn crystals belong to P2_1_2_1_2_1_ space group and have four molecules in the crystallographic asymmetric unit. The structure of Ms_orn was solved by molecular replacement using polyalanine models of bacterial Orns (PDB IDs: 2IGI, 1YTA, 1J9A, 2GBZ, and 3TR8) as templates in Phaser of CCP4 suite (63). The best solution was obtained with the polyalanine model of *E. coli* Orn (PDB ID: 2IGI) as the starting model. The model was built and refined using iterative cycles of COOT (64) and Refmac5 (65). The final structure of Ms_orn was validated using MolProbity (66) and the refinement statistics are summarized in Table 1.

Crystallization of 0.3 mM Ms_orn was also set up with 10-fold molar excess of GMP. Crystals were obtained in P4_1_2_1_2_1_ space group and diffracted to 2.73 Å. The structure was solved by molecular replacement using coordinates of high-resolution data as template. However, no unambiguous density for ligand was observed, so it was not considered further, and higher resolution structure of apo form at 1.87 Å is used to describe structural features of Ms_orn in the article.

Co-crystallization of Eco_orn was also attempted by incubating 1.0 μl of pre-mixed protein–ligand solution (7 mM GMP and 0.75 mM purified protein) in 20 mM Tris–HCl buffer, pH 8.5, 100 mM NaCl, and 5% glycerol with 1.0 μl of reservoir solution (0.1 M Hepes buffer, pH 7.5, 0.25 M sodium acetate, and 25% v/v PEG-3350) at 24 °C. Crystals were obtained after 1 week and cryo-protected in a solution containing 10% glycerol in addition to the crystal reservoir solution and flash frozen in liquid nitrogen before collecting diffraction data to 2.3 Å resolution. The structure of Eco_orn was solved by molecular replacement using coordinates of 2IGI but could not identify a bound ligand. The final structure was validated using MolProbity (66) and the refinement statistics are summarized in Table 1. The final refined structure of apo form of Eco_orn was hence used for comparative structural analysis with Ms_orn.

The atomic coordinates and structure factors of Ms_orn and Eco_orn have been deposited with the PDB with accession codes 7WIK and 7VH4, respectively.

### MD simulations

All atom MD simulations were carried out for Ms_orn using Gromacs, version 5.1.4 software package (67, 68). The coordinates of Ms_orn were first stripped of all crystallographic waters and ions, topologies were generated using CHARMM27 force field (69), and then solvated using SPC/E water model (70). The solvated system was minimized using 50,000 steps of steepest descent algorithm. The models were then separately subjected to position restrained canonically defined NVT (N: constant number of atoms, V: volume, and T: temperature) and NPT (N: constant number of atoms, P: pressure, and T: temperature) ensemble for 5 ns. The temperature of the system during NVT equilibrium was maintained at 300 K using Berendsen weak coupling method, and pressure in NPT equilibrium was maintained at 1 bar by using Parrinello–Rahman barostat. LINCS algorithm was used for constraining bonds (67). The long-range electrostatic interactions were calculated by using particle mesh Ewald (71). The van der Waals interactions were calculated through Lennard–Jones potential with cutoff of 0.1 nm. The production run of 500 ns was performed with integration time of 2 fs.

An all-atom MD simulation on Eco_orn was similarly carried out using Gromacs, version 5.1.4 for 500 ns before further analysis.

To monitor the effect of C-terminal tail of Ms_orn, *in silico* deletion of the tail from residues 181 to 198 or 186 to 198 was carried out to yield coordinates corresponding to Ms_ornΔC' or Ms_ornΔC. Further MD simulation of Ms_ornΔC' or Ms_ornΔC was carried out as described for full-length aforementioned Ms_orn. The generated MD trajectories were analyzed by *gmx rms* and *gmx gyrate* utilities of Gromacs to calculate rmsd_avg_ and *R*_g-avg_ values across the timescale of MD simulations of 500 ns. Graphical analysis of MD trajectories was done using Xmgrace.

### CD measurements

Far-UV CD measurements for Ms_orn, Ms_ornΔC, or Eco_orn were carried out with Jasco J-815 spectrophotometer equipped with a temperature controller (PTC-517). The CD instrument was continuously purged with nitrogen gas at a flow rate of 5 to 8 l/min and routinely calibrated with D-10 camphor sulfonic acid. Far-UV CD spectra were measured in the wavelength range of 250 to 195 nm with 0.2 mg/ml of respective protein in 20 mM Tris–HCl, pH 8.5, 100 mM NaCl, and 5% glycerol. CD measurements were carried out in a 1 mm path length cuvette at a scan rate of 100 nm/s, 1 nm bandwidth, and 1 s response time. An average of three consecutive scans, corrected by subtraction of buffer (used as blank), was used for each spectrum. Obtained raw CD data were converted into mean residue ellipticity at a wavelength [*θ*]_λ_ (deg cm^2^ dmol^−1^) by using the equation, [*θ*]_λ_ = *Mₒθ*_*λ*_*/*10*lc* (where, *Mₒ* is mean residue weight of a protein, *θ*_*λ*_ is observed ellipticity in mdeg at λ wavelength, *c* is concentration of protein (mg/ml), and *l* is cuvette path length in cm). All CD spectra measurements were carried out in triplicates.

### Thermal denaturation measurements by CD

Heat-induced denaturation of Ms_orn, Ms_ornΔC, or Eco_orn were carried out with 0.2 mg/ml of respective protein on a Jasco J-815 spectrophotometer. Denaturation was measured in the temperature range of 20 to 85 °C at a heating rate of 1 °C/min. Changes in CD signal were recorded at 222 nm, as a function of temperature. Obtained raw data were converted to mean residue ellipticity at [*θ*]_222_. Reversibility after heat denaturation was checked by cooling the denatured protein to 20 °C and then matching the spectrum with the spectrum taken before heating the protein. All thermal denaturation measurements were carried out in triplicates.

### Intrinsic fluorescence measurements

Fluorescence spectra of Ms_orn, Ms_ornΔC, or Eco_orn were measured with 0.2 mg/ml protein in 20 mM Tris–HCl, pH 8.5, 100 mM NaCl, and 5% glycerol using Jasco FP-6200 spectrofluorometer, equipped with an external thermostated water circulator to maintain constant sample temperature. The excitation was carried out at 295 nm followed by measurement of the emission spectra in the 300 to 400 nm wavelength range. All measurements were carried out in triplicates.

### Gdm-HCl–induced denaturation measurements by CD and fluorescence

A stock solution of 7 M Gdm-HCl was prepared in 20 mM Tris–HCl, pH 8.5, 100 mM NaCl, and 5% glycerol, and its concentration was determined by refractive index measurements. Equilibrium unfolding of Ms_orn, Ms_ornΔC, or Eco_orn induced by Gdm-HCl was monitored by far-UV CD as well as by intrinsic fluorescence. About 0.2 mg/ml of respective protein was incubated with different concentrations of Gdm-HCl between 0 to 3-4 M in several experimental runs for 2 to 3 h before spectral measurements. The denaturation curves with Gdm-HCl were finally carried out between 0 and 6 M of the denaturant in two independent runs, and the transition curves of Ms_orn, Ms_ornΔC, and Eco_orn were analyzed to estimate the thermodynamic parameters Δ*G*_I_ (Δ*G* associated with transition I) and Δ*G*_II_ (Δ*G* associated with transition II) using the following equations,

(1) $$\Delta G_{I}=-RT\;\ln\frac{y-y_{N}}{y_{X}-y}$$

(2) $$\Delta G_{\text{II}}=-RT\;\ln\frac{y-y_{X}}{y_{U}-y}$$

where *T* is temperature in Kelvin, *R* is universal gas constant, and *y*_N_, *y*_X_, or *y*_U,_ respectively, represent the properties of the protein molecules in the native, intermediate, or unfolded state at the same [Gdm-HCl] in which *y* (observed property of protein) was measured. A linear plot of Δ*G*_I_ or Δ*G*_II_ *versus* [Gdm-HCl] was obtained, enabling estimation of Δ*G*_I_^0^ (value of Δ*G*_I_ at 0 M Gdm-HCl) associated with transition I and Δ*G*_II_^X^ (value of Δ*G*_II_ in the presence of Gdm-HCl, where X state existed) associated with transition II, using least square analysis according to the equations,

(3) $$\Delta G_{I}=\Delta G_{I}^{0}-m_{I}\;\left[\text{Gdm}-\text{HCl}\right]$$

(4) $$\Delta G_{\text{II}}=\Delta G_{\text{II}}^{X}-m_{\text{II}}\left[\text{Gdm}-\text{HCl}\right]$$

### ANS measurements

The stock solution of ANS was prepared in water, and its concentration was determined using value of 5000 M^−1^ cm^−1^ for molar absorption coefficient at 350 nm (72). For ANS fluorescence measurements, the protein sample was incubated with 25-fold molar excess of ANS, for 2 to 3 h in the dark. Emission spectra were collected in the wavelength range of 400 to 600 nm after excitation at 360 nm. For all fluorescence measurements, quartz cuvette of 10 mm path length was used with excitation and emission slits, both set at 5 nm bandwidth. All experiments were repeated twice before plotting.

### Growth of *M. smegmatis* knockout and complemented strains

A knockout of *orn* in *M. smegmatis* was generated by a suicidal vector strategy followed by homologous recombination method (PB and BT [unpublished observations]). *M. smegmatis* mc^2^155 (WT), knockout strain (*orn*-depleted strains, Δ*orn*), and complemented strains (Δ*orn/orn*, Δ*orn/ornΔC*, *and* Δ*orn/ornΔC′*), complemented with clones in pTC0X1L, expressing proteins corresponding to full length (*orn*), 1 to 185 residues (*ornΔC*) or 1 to 180 residues (*ornΔC′*), were grown in Difco Middlebrook 7H9 broth supplemented with 0.25% glycerol, 0.05% Tween-80 and 0.4% glucose or on 7H9 medium containing 1.5% agar at 37 °C with constant shaking. Growth was monitored by measuring optical density at 600 nm using Bioscreen growth curve analyzer. Growth experiments were done for two independent biological replicates. All growth experiments with WT and knockout strain (Δ*orn*) were with an empty vector control (pTC0X1L).

### Growth under stress conditions

For UV stress, serial dilutions of WT, Δ*orn*, Δ*orn/orn*, Δ*orn/ornΔC*, and Δ*orn/ornΔC′* were spotted on 7H9 medium containing 1.5% agar plates and irradiated under UV light for 15 min. The plates were incubated at 37 °C in the dark for 2 to 3 days. For oxidative stress, WT, Δ*orn*, Δ*orn/orn*, Δ*orn/ornΔC*, and Δ*orn/ornΔC′* were grown in 7H9 broth to early log phase to a cell density of 0.2. Strains were incubated with different concentrations of H_2_O_2_ for 3 h. Cells were then serially diluted and spotted on 7H9 medium containing 1.5% agar and incubated at 37 °C for 2 to 3 days.

Growth under oxidative stress was also monitored by first growing WT, Δ*orn*, Δ*orn/orn*, Δ*orn/ornΔC*, and Δ*orn/ornΔC′* in 7H9 medium at 37 °C until an absorbance of ∼1.0 at 600 nm with constant shaking. The respective strains were then diluted 1:100 with fresh medium and 100 μl of each diluted culture was inoculated separately into honeycomb 100-well plates with varying concentrations of H_2_O_2_ (10, 5, 2.5, 1.25, and 0.625 mM H_2_O_2_). Growth was then monitored using Bioscreen growth curve analyzer every 3 h. The experiment was performed with two independent biological replicates.

### RNA isolation and qRT–PCR

About 10 ml cultures of WT, Δ*orn*, Δ*orn/orn*, Δ*orn/ornΔC*, and Δ*orn/ornΔC′* were grown to stationary phases (optical density of ∼1.0 at 600 nm), and RNA was isolated using TRIzol method. RNA was quantified by measuring absorbance at 260 nm and quality was checked with absorbance at 260 nm/absorbance at 280 nm and absorbance at 260 nm/absorbance at 230 nm ratios and on a 2% agarose gel. To synthesize complementary DNA, 1.5 μg RNA was first treated with DNase at 37 °C, followed by complementary DNA synthesis using SuperScript IV first-strand synthesis system (Invitrogen) and random hexamers, according to the protocol provided by the manufacturer. SYBR Green (Applied Biosystems) was used as indicator dye for qRT–PCR. The RNA levels were normalized with respect to the *rpoC* gene of *M. smegmatis* (encoding β′ subunit of RNA polymerase). The experiments were performed for three biological replicates, and each experiment was performed in three technical replicates.

### Western blotting for detection of Ms_orn in complemented strains

Complemented strains with clones expressing proteins corresponding to full-length Ms_orn (*orn*), 1 to 185 residues (*ornΔC*) or 1 to 180 residues (*ornΔC′*) with an N-terminal His_6_ tag were grown in 400 ml 7H9 medium for 24 h at 37 °C. Cells were harvested and respective protein from indicated strain was purified using Ni–NTA affinity chromatography as described for Ms_orn and concentrated to 100 μl. About 30 μl of protein aliquot was separated on 15% SDS-PAGE and electro-transferred to a nitrocellulose membrane. The membrane was blocked with 4% bovine serum albumin dissolved in 1× PBS for 2 h at room temperature and incubated overnight with 1:3000 dilution of mouse anti–His tag antibody at 4 °C. After washing the membrane two times with 1× PBS with Tween, followed by 1× PBS, the membrane was incubated with 1:1500 dilution of antimouse horseradish peroxidase–conjugated secondary antibody at room temperature for 3 h. The membrane was washed again with 1× PBS with Tween-20 and 1× PBS and incubated with chemiluminescent substrates. The developed membrane blot was imaged using ImageQuant LAS 500 (GE). Prestained protein ladder was used for molecular weight estimation.

### Enzyme activity assay

Phosphatase or PDE activity of Ms_orn, Ms_ornΔC or Eco_orn was tested using pNPP or bNPP as respective substrates. The reactions were carried out in separate 100 μl reaction mixes containing 4 μM of purified protein and 200 μM substrate in 20 mM Tris–HCl, pH 8.5, 100 mM NaCl, and 5 mM MgCl_2_. The reactions were incubated at 37 °C, and release of p-nitrophenol was monitored colorimetrically at λ_405nm_ using Tecan infinite 200 Microplate reader. Activity assay was performed for two biological replicates and each replicate was performed in triplicate.

## Data availability

All data described in the article are available from the authors on request. The atomic coordinates and structure factors of Ms_orn and Eco_orn have been deposited with the PDB with accession codes 7WIK and 7VH4, respectively.

## Supporting information

This article contains supporting information (73).

## Conflict of interest

The authors declare that they have no conflicts of interest with the contents of this article.
