# Supplementary material for: Three-dimensional structure of a mycobacterial oligoribonuclease reveals a unique C-terminal tail that stabilizes the homodimer
Source: J Biol Chem. 2022 Oct 14;298(12):102595. doi: 10.1016/j.jbc.2022.102595 (PMC9676404; doi:10.1016/j.jbc.2022.102595)
Supplement: Supporting information [file mmc1.docx]

**Supporting information**

**Three-dimensional structure of a mycobacterial oligoribonuclease reveals a unique C-terminal tail that stabilizes the homodimer**

**Pooja Badhwar^1,2^, Sabab Hasan Khan^1^ and Bhupesh Taneja^1,2*^**

^1^CSIR-Institute of Genomics and Integrative Biology (CSIR-IGIB), New Delhi-110025 India; ^2^Academy of Scientific and Innovative Research (AcSIR), Ghaziabad-201002, India

*btaneja@igib.res.in

**Table S1: List of primers used**

| S. no. | Primer | Sequence (5̍-3̍ ) |
| --- | --- | --- |
| 1. | Ms_orn cloneA-F | TATAGGATCCGTGCGAATACTGTGGATCTTC |
| 2. | Ms_orn cloneB-F | TATAGGATCCGTGCGAGACGAACTGGTGTG |
| 3. | Ms_orn-R | TATACTCGAGTTAAGCCGAATCGGTATCCG |
| 4. | Ms_ornΔC’-R | TATACTCGAGTTACGGGACGAACGCCGTG |
| 5. | Ms_ornΔC-R | TATACTCGAGTTAAGAAGGCCCGGGCTGC |
| 6. | Eco_orn-F | TATAGGATCCATGAGTGCCAATGAAAACAAC |
| 7. | Eco_orn-R | TATAAAGCTTTTACAGCTTGATAAAATGCTCG |
| 8. | *orn*-F | TATACATATGCACCATCATCATCATCACGTGCGAATACTGTGGATCTTC |
| 9. | *orn*-R | TATAGAATTCTTAAGCCGAATCGGTATCCGCCGA |
| 10. | *ornΔC­*-R | TATAGAATTCTTAAGAAGGCCCGGGCTGCGG |
| 11. | *ornΔC­’*-R | TATAGAATTCTTACGGGACGAACGCCGTGGC |
| 12. | rpoC-F | CTGCCGAAGAGCTATCCGTT |
| 13. | rpoC-R | TTGAGCTTGTCGACGGTCTG |
| 14. | *orn*RT-F | GCTCGACGATTACCTGCACT |
| 15 | *orn*RT-F | CGAGACCTTTCTCCGGTTGG |

**Table S2: List of H-bonds between dimeric interfaces of Ms_orn and Eco_orn (as predicted by PDBePISA)^a^**

|  | **Ms_orn** | | |  | **Eco_orn** | | |
| --- | --- | --- | --- | --- | --- | --- | --- |
| **Sr. No.** | **Subunit B** | **Distance (Å)** | **Subunit A** |  | **Subunit B** | **Distance (Å)** | **Subunit A** |
| 1. | Arg 141 [NE] | 2.89 | Arg 2 [O] |  | Arg 143 [NH1] | 3.06 | Asn 6 [O] |
| 2. | Trp 142 [NE1] | 2.84 | Ala 32 [O] |  | Trp 144 [NE1] | 2.98 | Ala 35 [O] |
| 3. | Ser 134 [OG] | 2.74 | Met 129 [O] |  | Arg 143 [NH2] | 3.50 | Lys 101 [O] |
| 4. | Asp 131 [N] | 2.93 | Ser 134 [OG] |  | Thr 136 [OG1] | 2.97 | Tyr 131 [O] |
| 5. | Arg 128 [NH1] | 2.91 | Glu 137 [OE2] |  | Arg 130 [NH1] | 2.66 | Glu 139 [OE2] |
| 6. | Val 179 [N] | 3.55 | Tyr 143 [OH] |  | Lys 180 [NZ] | 2.88 | Glu 176 [O] |
| 7. | Val 179 [N] | 2.89 | Ala 177 [O] |  | Lys 180 [N] | 2.89 | Phe 178 [O] |
| 8. | Tyr 143 [OH] | 2.57 | Val 179 [O] |  | Lys 145 [NZ] | 2.66 | Lys 180 [O] |
| 9. | Arg 2 [O] | 2.90 | Arg 141 [NE] |  | Asn 6 [O] | 2.98 | Arg 143 [NH1] |
| 10. | Asp 3 [O] | 3.08 | Arg 141 [NE] |  | Ala 35 [O] | 3.01 | Trp 144 [NE1] |
| 11. | Glu 4 [O] | 3.79 | Arg 141[NH2] |  | Lys 101 [O] | 3.67 | Arg 143 [NH2] |
| 12. | Ala 32 [O] | 2.81 | Trp 142 [NE1] |  | Tyr 131 [O] | 3.05 | Thr 136 [OG1] |
| 13. | Met 129 [O] | 2.72 | Ser 134 [OG] |  | Glu 139 [OE1] | 2.68 | Arg 130 [NH1] |
| 14. | Ser 134 [OG] | 2.95 | Asp 131 [N] |  | Glu 176 [O] | 3.26 | Lys 180 [NZ] |
| 15. | Glu 137 [OE2] | 2.79 | Arg 128 [NH1] |  | Phe 178 [O] | 2.90 | Lys 180 [N] |
| 16. | Tyr 143 [OH] | 3.57 | Val 179[N] |  | Lys 180 [O] | 2.84 | Lys 145 [NZ] |
| 17. | Ala 177 [O] | 2.86 | Val 179 [N] |  |  |  |  |
| 18. | Val 179 [O] | 2.53 | Tyr 143 [OH] |  |  |  |  |
| 19. | Gln 181 [NE2] | 2.75 | Trp 142 [O] |  |  |  |  |
| 20. | Arg 145 [NH1] | 3.12 | Pro 184 [O] |  |  |  |  |
| 21. | Trp 142 [O] | 2.52 | Gln 181 [NE2] |  |  |  |  |
| 22. | Gly 183 [O] | 3.69 | Arg 145 [NH2] |  |  |  |  |
| 23. | Pro184 [O] | 2.94 | Arg 145 [NH2] |  |  |  |  |

^a^H-bonds that are formed by C-terminal α10 helix in Ms_orn are highlighted.


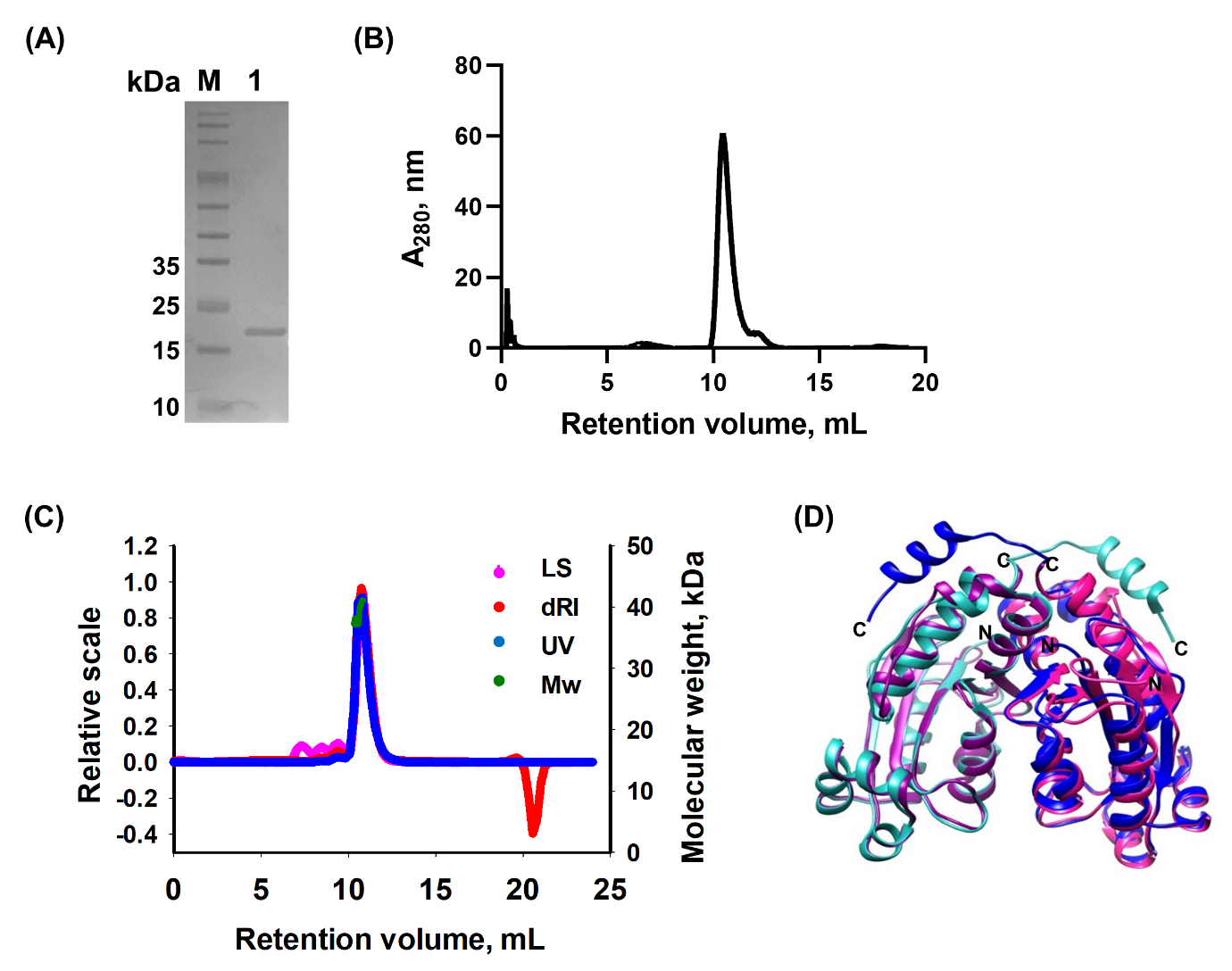


**Figure S1**: **Purification and size estimation of Eco_orn.** **(A)** Electrophoretic profile of purified Eco_orn on a 10% SDS-PAGE indicates a band of high purity. **(B)** SEC profile of Eco_orn on superdex-75 column; the protein elutes at 10.5 ml corresponding to a dimer with an estimated M_w_ of 40.6 kDa. Estimated M_w_ was calculated using calibration curve with molecular weight standards shown in Figure 1B. **(C)** SEC-MALLS of Eco_orn showing signals for LS, dRI and UV (280 nm) and a calculated M_w_ of 38.6 kDa (D) Superposition of Eco_orn (magenta and pink subunits) over Ms_orn (cyan and blue subunits), showing the absence of extended C-terminal tail in Eco_orn. A r.m.s.d. of 1.134 Å for 347 aligned residues is obtained for superposition of Ms_orn and Eco_orn.


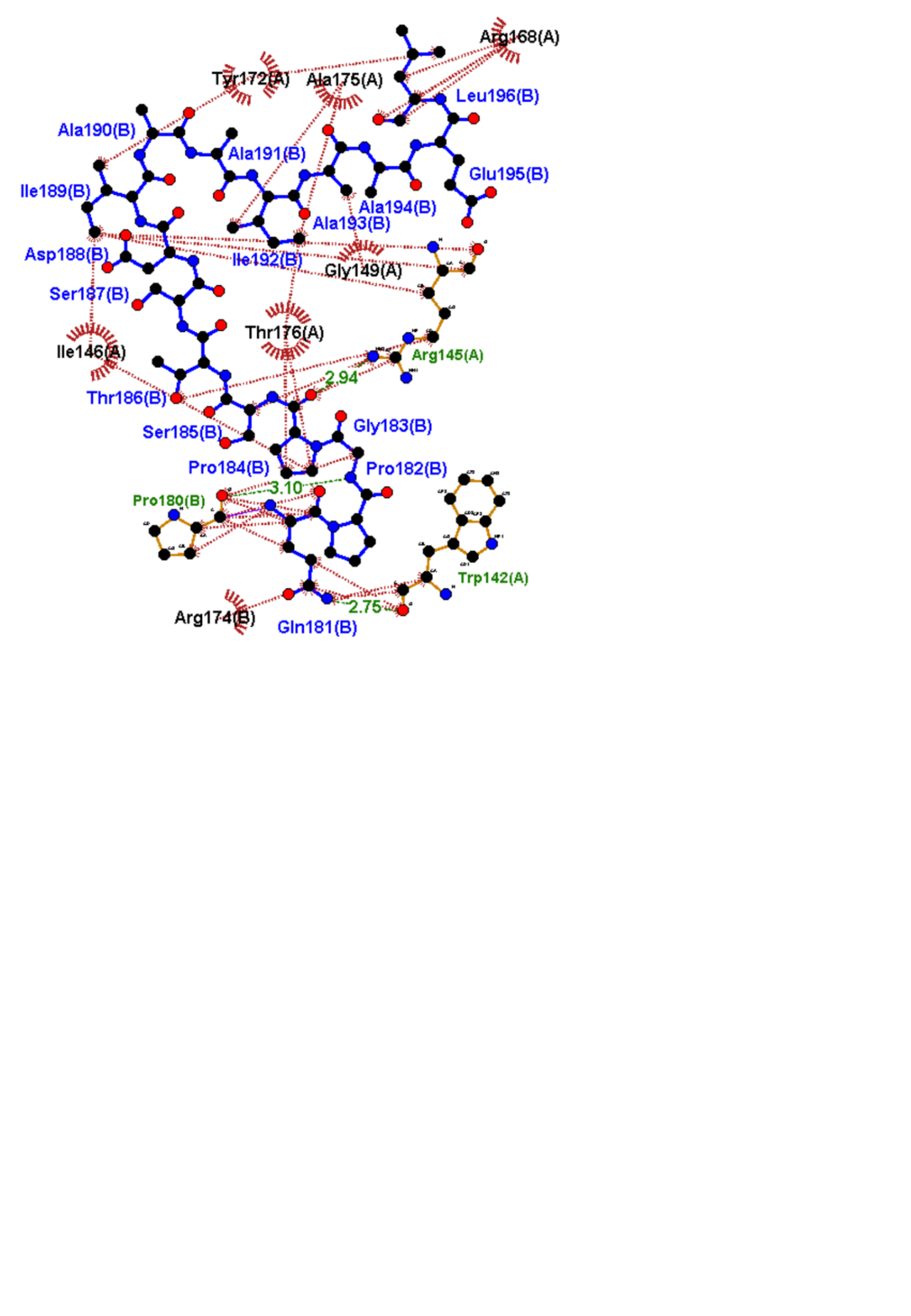


**Figure S2:** **Interactions between the C-terminal tail and the main domain of the two subunits**. Interactions between residues of the C-terminal tail of one subunit (chain B) and the main domain of other subunit (chain A), (H-bonds in green and hydrophobic interactions in red) as estimated using Ligplot [76].


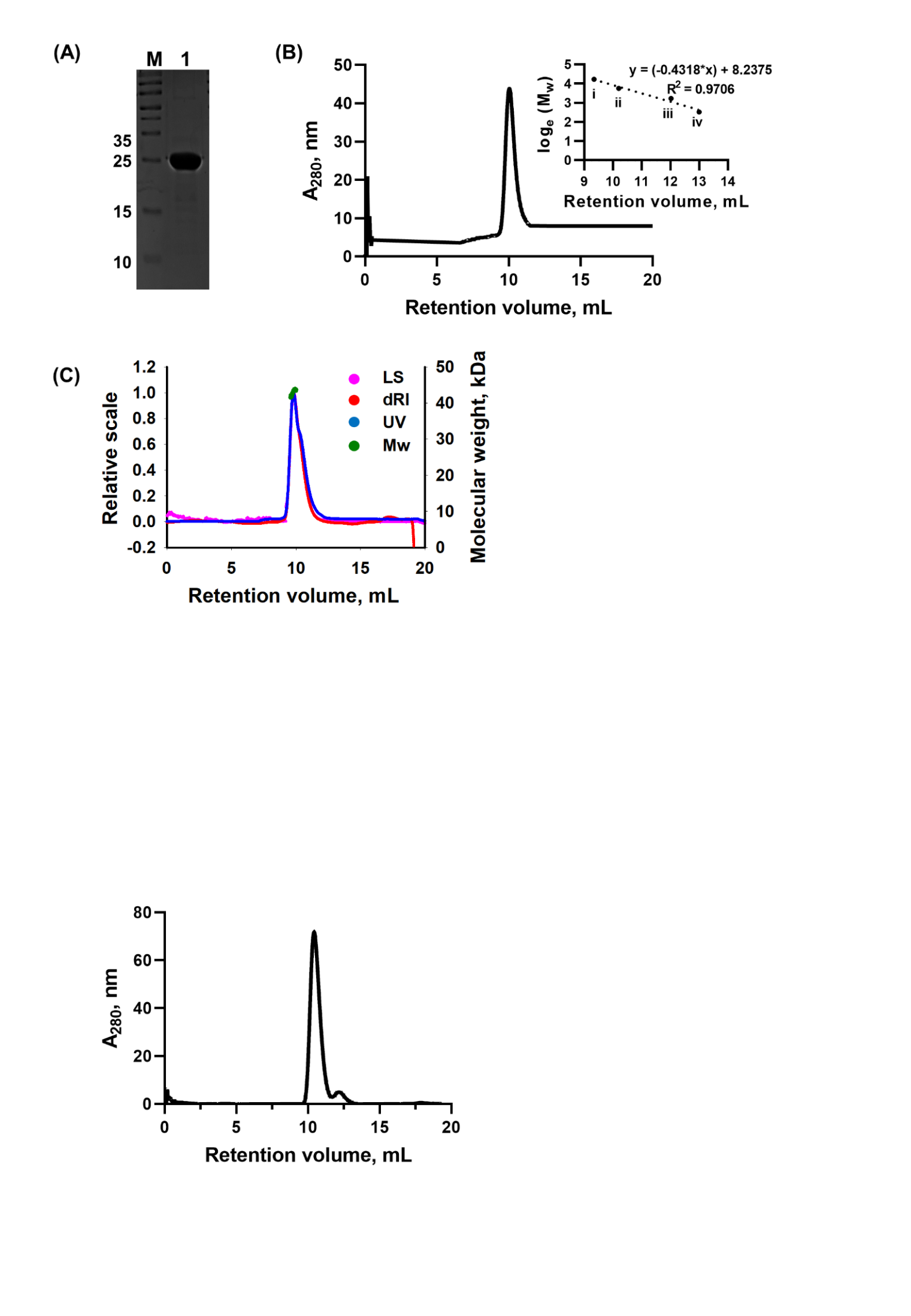


**Figure S3: SEC profile of Ms_ornΔC**. Ms_ornΔC elutes as a dimer on a superdex-75 column, at 10.4 ml (estimated M_w_ 41.6 kDa). The estimated M_w_ was calculated using calibration curve with molecular weight standards shown in Figure 1B.


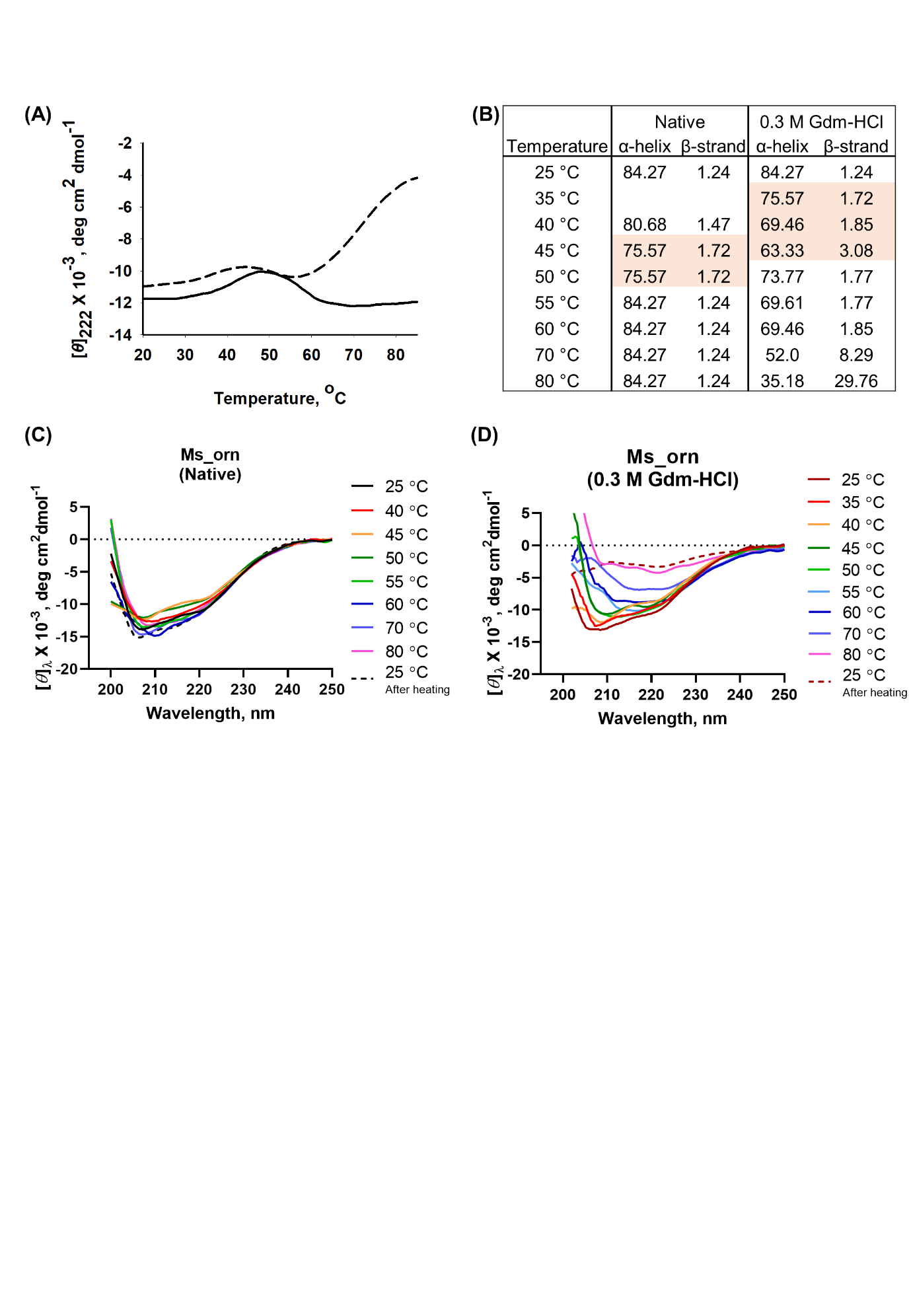


**Figure S4: Change in local conformation during thermal denaturation of Ms_orn**. (A) Thermal denaturation profile of Ms_orn in native buffer condition (solid lines) and in presence of 0.3 M Gdm-HCl (dashed lines). (B) Measurement of α-helix and β-sheet content in Ms_orn at different temperatures, using K_2_D_2_ program of Dichroweb server. (C) Far-UV CD spectra of Ms_orn in native buffer conditions taken at different temperatures between 25°C and 80°C. (D) Far-UV CD spectra of Ms_orn in presence of 0.3 M Gdm-HCl at temperatures ranging from 25°C to 80°C.


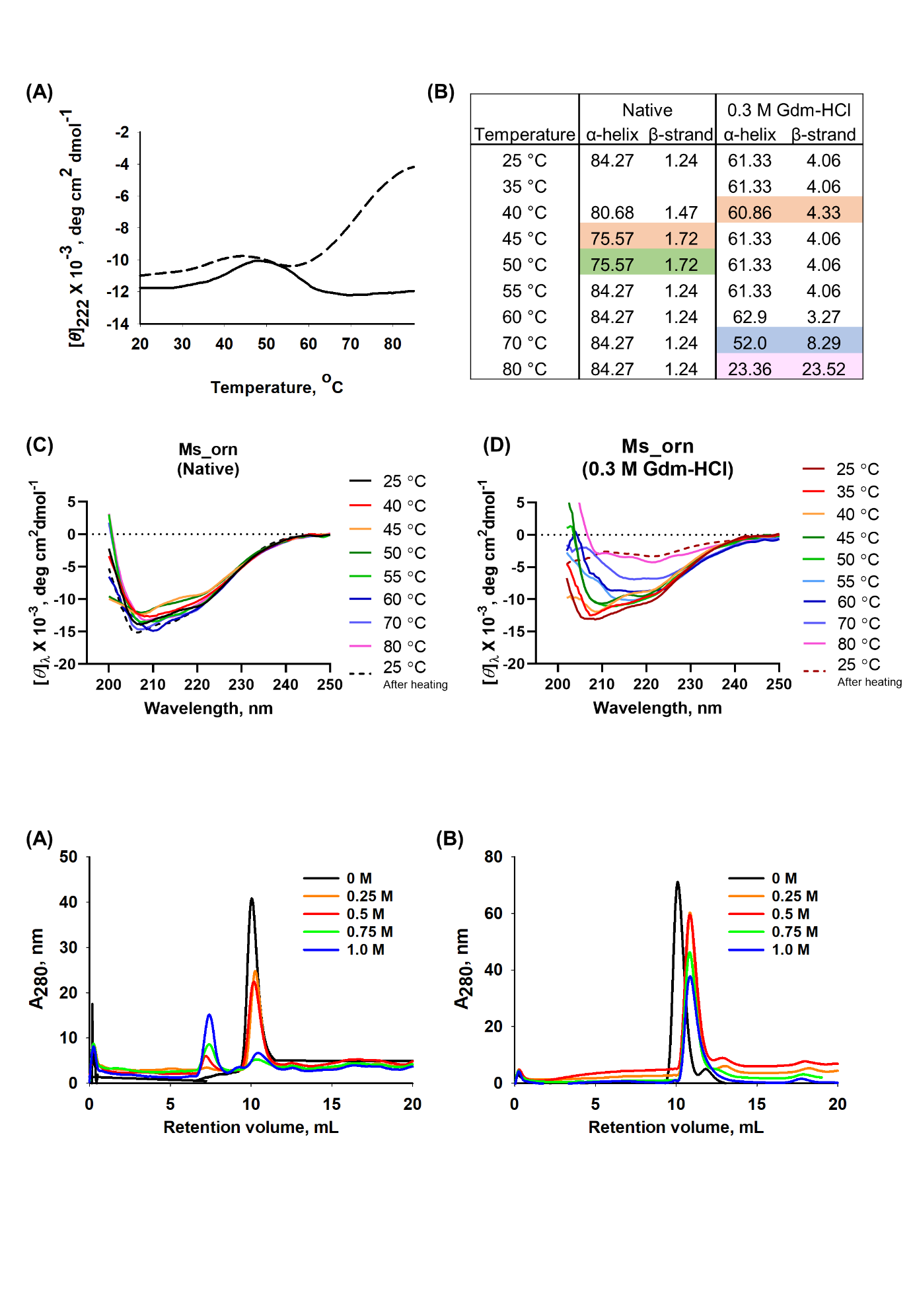


**Figure S5:** **SEC profile of Ms_orn as a function of [Gdm-HCl]**. SEC was carried for Ms_orn over Superdex-75 column to estimate its oligomeric states in presence of increasing concentration of Gdm-HCl.


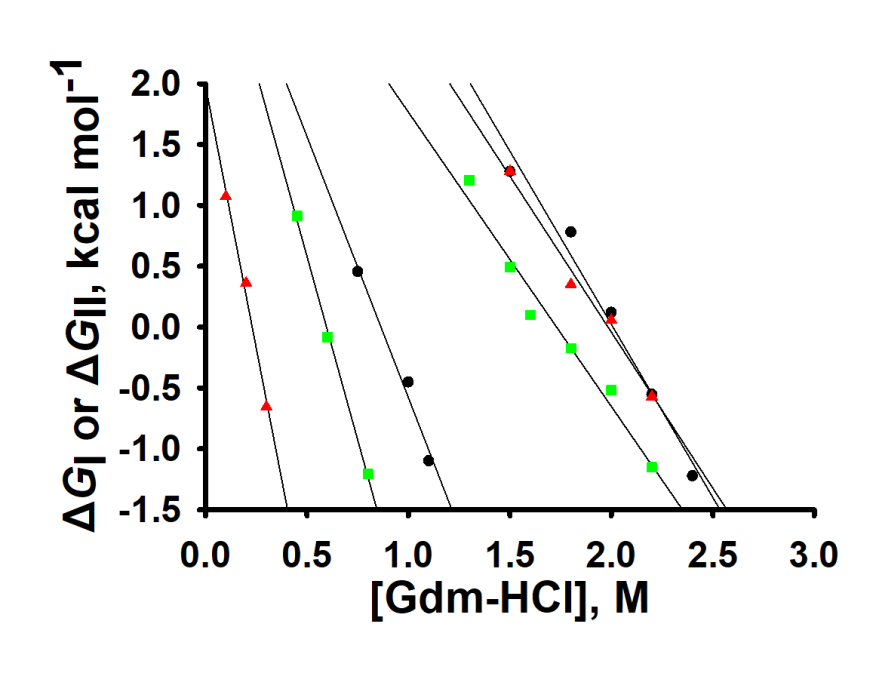


**Figure S6: Δ*G*_I_ or Δ*G*_II_ plots of Gdm-HCl-induced denaturation**. Δ*G*_I_ or Δ*G*_II_ plots of Gdm-HCl-induced denaturation of Ms_orn (black), Ms_ornΔC (red) and Eco_orn (green), as obtained from [*θ*]_222_ measurements.


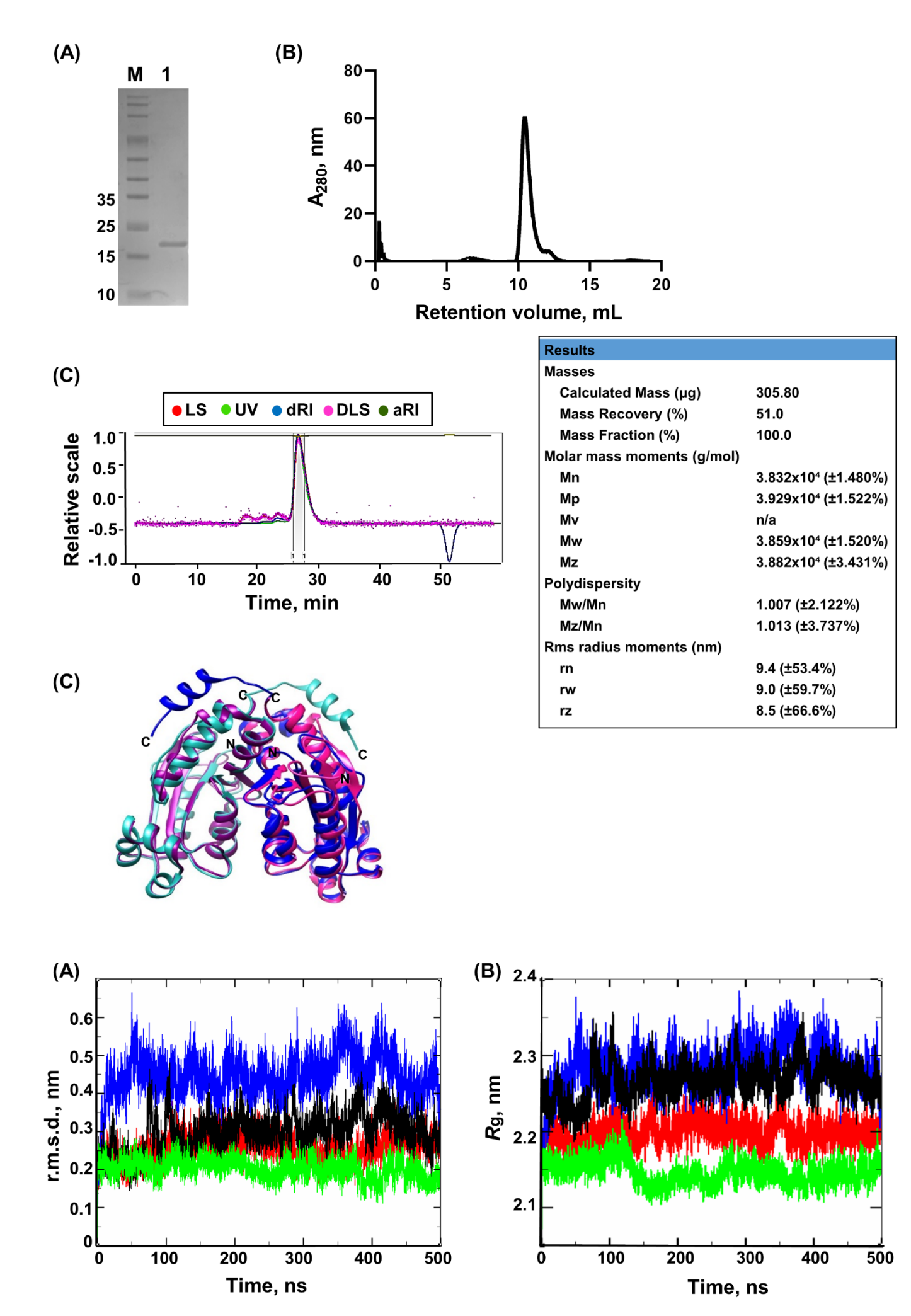


**Figure S7:** **MD simulation studies of Eco_orn**. (A) r.m.s.d. and (B) *R*_g_ plot of Eco_orn (green) are plotted. Respective r.m.s.d. and *R*_g_ plots of Ms_orn (black), Ms_ornΔC (red) and Ms_ornΔC’ (blue) curves are reproduced in (A) and (B) from Fig. 8A and B, respectively, for comparison with Eco_orn.
